# iCARE Self-Guided Digital Intervention for Postpartum Depression in Danish Mothers: Formative Research Using User-Centered Design

**DOI:** 10.2196/73948

**Published:** 2026-05-13

**Authors:** Maria Marti-Castaner, Charlotte Thomsen, Anja Friis Elliott, Emilie Holt, Sejla Husedzinovic, Kathrine Bang Madsen, Trine Munk-Olsen

**Affiliations:** 1 Department of Public Health Faculty of Health and Medical Sciences University of Copenhagen Copenagen Denmark; 2 Department of Clinical Research, Odense University Hospital, Research Unit of Digital Psychiatry University of Southern Denmark Odense, South Denmark Denmark; 3 Department of Clinical Research Research Unit of Child and Adolescent Psychiatry University of Southern Denmark Odense Denmark

**Keywords:** postpartum depression, mother, digital intervention, cognitive therapy, user-centered design, stepped care

## Abstract

**Background:**

Postpartum depression (PPD) is a major public health concern. Despite advancements in treatment, many barriers to accessing care remain. There has been a growing interest in digital interventions for the prevention and treatment of PPD. However, for mothers with mild and moderate symptoms of depression, there is a limited offer of self-guided internet-based interventions developed with user input and with considerations on how to integrate the intervention into stepped care models for PPD.

**Objective:**

The aim of this study was (1) to describe the process of the design and development of iCARE, a self-guided digital psychological intervention for mothers with mild and moderate symptoms of PPD in Denmark, (2) present the program’s theory illustrated by a logic model, and (3) explore its initial usability and prospective acceptability.

**Methods:**

Applying user-centered design methods, the intervention development followed six steps: (1) a literature review to identify evidence‑based therapeutic components of self‑guided interventions for PPD, (2) interviews with women with lived experience of PPD and group discussions with mental health experts and home‑visiting providers to identify user needs, (3) iterative design and content development with stakeholder feedback in collaboration with the Department of Digital Psychiatry, (4) prototype testing using think‑aloud usability sessions and interviews with 5 mothers, (5) a group cognitive walkthrough with mental health experts, and (6) final refinement and implementation of the iCARE program with developers and designers.

**Results:**

Initial interviews with mothers and maternal health care providers emphasized the importance of a digital intervention offering timely psychoeducation, coping strategies, and pathways to in-person care while addressing the diversity of expressions of PPD symptoms. Stakeholders recommended a flexible program, multimodal content, and integration into maternal care systems with community health nurses supporting engagement and participation. The prototype was designed to be user-centered, engaging, and with multiple interactive features. It included components on psychoeducation, cognitive exercises grounded in cognitive behavioral therapy, acceptance and commitment principles, and mood-monitoring. The prototype was designed to be user-centered and engaging, with interactive features and components on psychoeducation, cognitive exercises grounded in cognitive behavioral and acceptance and commitment principles, and mood-monitoring. Prototype testing indicated high prospective acceptability and led to refinements across 6 themes: appropriateness of content; motivation and engagement; inclusivity and gender representation; clarity of instructions and data use; understanding of therapeutic method; and usability, layout, and navigation.

**Conclusions:**

iCARE is a self-guided internet-based psychological intervention for mothers with mild and moderate symptoms of PPD in Denmark. It was developed with user input by using qualitative methods, user-centered design, and psychological theory. Further research is needed to evaluate the feasibility and effectiveness of the program in a randomized controlled trial and its integration into maternal health care models such as universal PPD screening and home-visiting.

## Introduction

### Background

Postpartum depression (PPD) affects 10% to 15% of mothers globally [1]. Universal PPD screening is increasingly common in health care, but its effectiveness depends on systems that ensure timely interventions for those who screen positive [2,3]. Stepped care models address this gap by pairing systematic screening with risk assessment and tailored interventions. [4]. Despite treatment advancements, access to care remains limited due to stigma, service fragmentation, resource shortages, high costs, and logistical challenges [2,3]. Persistent barriers highlight the need for accessible, innovative approaches integrated into existing stepped care models. To enhance the stepped care model for PPD, this study explored how formative research can facilitate the development of a self-guided digital mental health intervention (DMHI; iCARE) for mothers with mild-to-moderate symptoms of PPD living in Denmark.

DMHIs offer a promising alternative to traditional face-to-face care [5]. Over the past decade, DMHIs for PPD prevention and treatment have shown modest effects in reducing short-term depressive and anxiety symptoms [6]. These typically include cognitive behavioral therapy (CBT), psychoeducation, mindfulness, or combinations thereof, with psychotherapeutic components proving more effective than psychoeducation alone. DMHIs vary in delivery, from self-guided to blended models (combining digital tools with in-person or telehealth support), and technology (app- or web-based) [7]. While self-guided interventions can broaden access, challenges with engagement and retention often limit their implementation, effectiveness, and scalability [8]. Addressing these issues through improved design, content, and delivery is crucial for enhancing their use and impact [9].

Early work to inform the development of iCARE included an examination of existing self‑guided digital interventions for PPD. Prior programs, such as MUMentum [10], MomMoodBooster [11], and Happy Mother [12], demonstrate that digital interventions can incorporate cognitive‑behavioral strategies and show promising effects, particularly when developed through iterative user feedback. However, most self‑guided interventions remain outside routine maternal care.

### The iCARE Study

Against this background, this study aimed to develop iCARE, a self-guided digital psychological intervention for mothers in Denmark with mild-to-moderate PPD, designed for integration into universal postnatal care. Denmark provides an ideal context given its digitalized health care system, established internet-based treatments for depression, and universal nurse home-visiting program, which includes PPD screening at the 8-week visit using the Edinburgh Postnatal Depression Scale (EPDS). While maternal health care providers (MHCPs) can offer extra visits, group sessions, or referrals, two-thirds of mothers who screen positive still lack appropriate care [13] and less than 50% of the municipalities’ MHCPs do not have the option to refer mothers directly to some kind of treatment for PPD within the municipality [14]. Integrating a self-guided DMHI could help close these gaps and inform similar care models internationally.

A self-guided format was chosen for its scalability and suitability for mothers facing stigma, time constraints, or logistical barriers, but also because some women might prefer the independence and privacy of this option. Although guided or blended interventions often yield stronger effects, they require therapist time and organizational capacity that are limited in Danish maternal care, making a self-guided approach the most practical option.

Using an iterative, user-centered design, this study also assessed initial usability and prospective acceptability, defined as anticipated willingness to engage and perceived relevance before implementation, based on feedback from women with lived experience of PPD. Two questions guided this research: (1) what are the preferences and recommendations of mothers, MHCPs, and experts for a self-guided digital intervention integrated into Denmark’s maternal care system? (2) What is the initial usability and prospective acceptability of the iCARE prototype? These questions shaped the iterative development and refinement of iCARE.

## Methods

### Study Design

**Figure 1:**
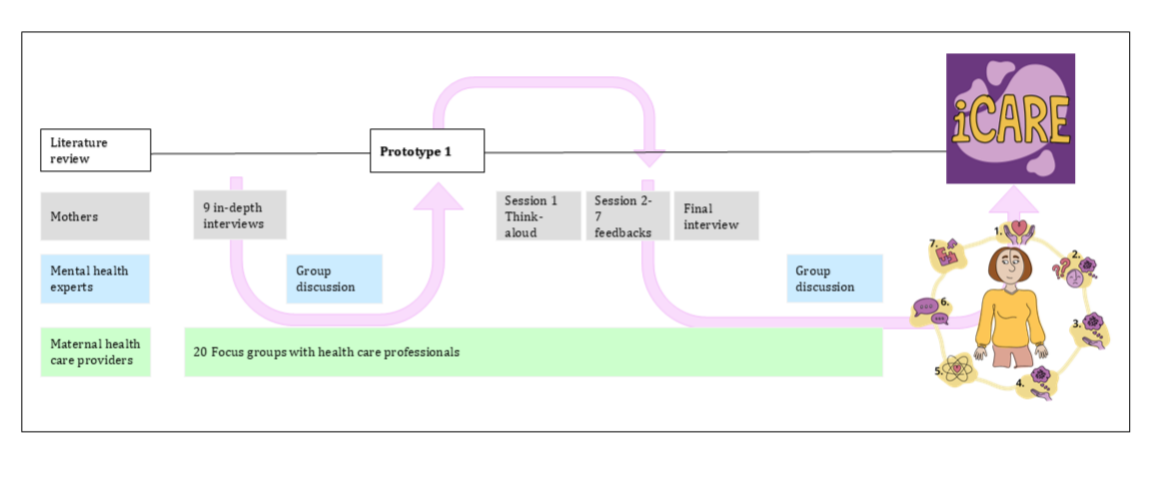
Flowchart of the iCARE development process.

We used an iterative intervention development process (Figure 1) guided by the Medical Research Council (MRC) framework for the development of complex interventions [15]. The MRC framework emphasizes establishing a theoretical foundation, integrating formative research, refining prototypes, and preparing an intervention for feasibility testing. Using user‑centered design principles [16], the development of iCARE proceeded through 6 steps described below. Subsequent subsections provide detailed recruitment, data collection, and analytic procedures.

#### Step 1: Literature Review

To inform the intervention’s theoretical foundation and therapeutic components, we conducted a literature review of minimally guided or self‑guided digital interventions for PPD (PROSPERO CRD42023482966). We included studies evaluating web‑ or app‑based programs for mothers with elevated PPD symptoms. Information was extracted on intervention structure, therapeutic strategies (eg, CBT, Acceptance and Commitment Therapy [ACT], and psychoeducation), engagement features, delivery formats, and implementation considerations. Findings informed the preliminary logic model and early decisions about iCARE’s content, therapeutic approach, format, and multimodal delivery.

#### Step 2: Interviews and Group Discussions With Stakeholders

We conducted formative qualitative work with 3 stakeholder groups: mothers with lived experience of PPD, MHCPs, and mental health experts. These activities explored mothers’ needs and preferences, MHCPs’ practices and perspectives on integration into maternal care, and experts’ recommendations on therapeutic content and structure. Insights from these interactions directly informed the logic model and early design requirements. Detailed recruitment procedures and data collection methods are described below.

#### Step 3: Iterative Design and Content Development

Design work was conducted with the Department of Digital Psychiatry. Early prototypes were inspired by an existing self‑guided CBT‑based digital intervention for depression developed at the Center for Digital Psychiatry, which includes 9 modules on psychoeducation, behavioral activation, cognitive restructuring, and relapse prevention, using videos, text, interactive exercises, and digital diaries. This model informed iCARE’s initial format, module structure, and some CBT‑based elements (eg, the cognitive-behavioral diamond). These components were then iteratively adapted and expanded using feedback from mothers, MHCPs, and experts to ensure relevance to postpartum experiences.

#### Step 4: Prototype Testing With Mothers

We conducted think‑aloud usability testing with 5 mothers from Step 2. Participants completed an introductory module while verbalizing their reactions, then independently explored the remaining modules over 3 weeks before a follow‑up interview. These combined data provided insight into usability, clarity, and prospective acceptability. Full procedures are described in the “Data Collection” section.

#### Step 5: Cognitive Walkthrough With Mental Health Experts

An online group cognitive walkthrough with mental health experts assessed the clarity, therapeutic coherence, and usability of the prototype. Experts were asked to review full sessions and provide feedback on content, format, tone, and clinical safety. Their recommendations informed adjustments to language, examples, and user flow.

#### Step 6: Final Refinement and Implementation

Feedback from mothers, MHCPs, and experts was synthesized and used to refine the prototype’s content, structure, language, and interface. Revisions were implemented in collaboration with developers and designers, resulting in a finalized version prepared for feasibility testing.

The following sections describe participant recruitment, data collection procedures, and analytic approaches in detail.

### Participants

Data were collected between June 2023 and October 2024 and involved 3 stakeholder groups: mothers with lived experience of PPD, municipal MHCPs, and mental health experts in perinatal mental health and digital interventions. Mothers were recruited through advertisements on the social media platforms of “Sind og Fødsel” and in Facebook groups (Meta Platforms, Inc) for new parents. Mothers were Danish‑speaking, aged 28-41 years, and all had completed at least a high‑school level education. All had experienced PPD and/or anxiety within the past 2 years. Seven of the 9 mothers had only 1 child. MHCPs were identified via LinkedIn (Microsoft Corp) and contacted directly through municipal health teams using a combination of convenience and purposive sampling to ensure representation from municipalities with and without existing PPD support offers. Mental health experts were identified through the research team’s professional networks based on their expertise in maternal mental health, digital interventions, or complex intervention development. A summary of inclusion criteria and participants’ characteristics is presented in Table 1.

**Table 1:** Summary of the participants, inclusion criteria, and characteristics.

Participants	Inclusion criteria	Participant characteristics
Mothers with lived experience (n=9)	≥18 years Experienced PPD^a^ within the last 2 years Danish‑speaking Self‑reported not having active depressive symptoms	Age range: 28-41 years Has only 1 child (n=7) Experienced PPD and/or anxiety with first child (n=9) Education: high school or higher (n=9)
Municipal MHCPs^b^ (n=20 focus groups, 1 focus group per municipality)	All municipalities eligible	2-4 MHCPs per focus group Majority were child health nurses; a few were psychologists or family counsellors employed in the home‑visiting program
Mental health experts (n=8)	Expertise in perinatal mental health, digital mental health interventions, complex intervention design, or representation of experts by experience	2 directors of the patient organization “Sind og Fødsel” 1 psychiatrist 1 psychologist and global mental health researcher 1 clinical psychologist expert in maternal mental health and health interventions 1 clinical psychologist expert in digital interventions 1 researcher expert in complex interventions 1 psychologist expert in PPD and attachment‑based interventions

^a^PPD: postpartum depression.

^b^MHCP: maternal health care provider.

### Data Collection

#### Interviews With Mothers (Round I: Preprototype)

Initial interviews were conducted by 5 different researchers with health backgrounds and supervised by the first author (a clinical psychologist). Interviews lasted 45-90 minutes and followed a topic guide (Multimedia Appendix 1) developed and pilot tested by the research team. The guide was structured in two parts: (1) experiences with PPD, support preferences, and technology use, and (2) feedback on digital mental health interventions, including content, design, and usability. All interviews were audio-recorded and transcribed.

#### Prototype User Testing With Mothers (Round II)

After the first prototype of iCARE was developed, mothers from Round I were invited to participate in usability testing. Five of 9 women agreed to participate (4 declined due to time constraints). Data collection began with an online think‑aloud session [17] in which a researcher guided each participant through the introductory module while prompting them to verbalize their immediate reactions to the content, layout, and navigation. Participants were then given 3 weeks to review the remaining modules independently at their own pace. After each module, they completed a short questionnaire assessing clarity, perceived relevance, and ease of navigation. A final semistructured interview explored their overall experience, including usability, comprehensibility, emotional tone, and prospective acceptability. The guiding questions are included in Multimedia Appendix 2. The think‑aloud sessions provided real‑time, in‑moment feedback, whereas the follow‑up interviews captured broader reflections after full exposure to the prototype.

#### Focus Groups With MHCPs

Focus groups with MHCPs were conducted in-person or online and included between 2 and 6 participants per group, primarily child health nurses and, in some cases, psychologists or family counsellors working within the Danish home-visiting program. Discussions focused on PPD screening practices, existing care pathways, practical barriers and opportunities for implementation, and perceived relevance of a self‑guided program such as iCARE. The interview guide was inspired by the Normalization Process theory [18] (see interview guide in Multimedia Appendix 3) to elicit insights related to adoption and integration within routine care. All sessions were recorded and field notes were taken.

#### Discussions With Mental Health Experts

During the development of iCARE, we consulted with all experts twice. In the first meeting, we discussed the initial logic model based on findings from the literature review and first interviews with mothers, and the potential intervention structure and general content. Before the second meeting, experts were given access to the prototype to explore the different sessions. In the second online meeting, we presented the iCARE prototype and showcased specific components of the intervention, asking specific questions guided by cognitive walkthrough methodologies to obtain feedback about functionality and content of the intervention. Additionally, we had further discussions with one expert who provided several rounds of feedback to improve one of the sessions based on her expertise.

### Data Analysis

#### Overview

We used a combination of thematic analysis with rapid qualitative analysis (RQA) to respond to the project’s needs and the multiple data sources. This approach has been deemed efficient and appropriate for studies applying a user-centered design approach to intervention development [19]. Below, we detail the processes used to develop the intervention by combining and integrating different sources of data.

#### Interviews With Mothers (Round I: Preprototype)

In the first phase, we analyzed the transcripts from interviews with 9 mothers using thematic analysis [20]. This method entails coding data and iteratively developing a set of themes through several stages of data review and comparison. First, EH and SH worked together using thematic network analysis [21] to code the first 5 interviews and clustered codes into themes through interpretation of how they related and were linked to each other. This initial coding was first done inductively and then using Borghouts framework concerning barriers and facilitators for engagement in digital interventions [5]. In a second stage, the first author analyzed all interviews using thematic analysis and, in discussions with the research team, synthesized and reorganized the basic themes into 4 main themes and 32 basic themes to guide the development of the intervention. For each basic theme, the first author identified actions to be taken in the intervention. This step was discussed with the research team and directly informed the development of the intervention. This step specifically informed the content of each module (including examples and exercises), format and learning modalities, and integration into maternal care.

#### Prototype User Testing With Mothers (Round II)

All interviews were recorded and automatically transcribed. The second author took notes of the interviews concurrently, identified themes for improvement recommended by the mothers and subsequently discussed specific suggestions with the research group. Some surface changes were made immediately (ie, deleting a graph of a pacifier that was used to divide sections). Then, using RQA, the first author conducted a swift review of interview transcripts and identified actionable feedback across 3 domains: format (videos, audios, graphics, and text), structure (number of modules, progression, and time to complete), and content (clarity and acceptability of therapeutic components, exercises, and language). The second author also reviewed all transcripts and the 2 researchers discussed and resolved differences, clustering observations into 5 themes for improvement linked to the prospective acceptability of iCARE, which guide the presentation of results. After discussing the results with the whole research group, the intervention was revised based on these themes and collective decisions were made on which elements of the feedback would be revised and tested in the feasibility study, given time constraints (eg, video rerecordings were not possible at this stage). In a later iteration, the first author coded all data in Microsoft Excel, using the previously identified themes as a deductive framework. Each transcript was reviewed and quotes were entered into a structured spreadsheet with columns for themes, suggested changes, and implementation status. This matrix allowed systematic verification that no relevant themes or potential adaptations were missed during the rapid coding process and facilitated comparison across interviews and rounds.

#### Data From MHCPs and Mental Health Experts

During the discussions with mental health experts, detailed notes were taken. After each meeting, notes were discussed in the research team and RQA was used to identify any feedback related to the format, structure, content, and implementation of the intervention.

Focus groups with the MHCP informed the intervention’s tailoring and integration on a running basis as the focus groups took place over the entire development phase. Salient themes were identified along the way using RQA and choices were made to respond to feedback on the intervention and its implementation. However, an independent analysis of the focus groups will be reported separately.

Feedback from mental health experts and MHCP was integrated with the analysis of mothers’ responses to the prototype, which we describe in the “Results” section, and informed the refinement of iCARE.

### Ethical Considerations

As outlined by the Danish National Committee on Health Research Ethics, researchers are only required to seek ethical approval for projects involving experiments on live-born individuals, clinical drug trials, or studies using sensitive bioinformatics data [22]. Therefore, formal ethical approval was not necessary for this study. Nonetheless, the study adhered to the principles of the Declaration of Helsinki and the International Ethical Guidelines for Health-related Research. The project was approved by the Regional Data Protection Centre in Psychiatry, Region of Southern Denmark (23/15327), the University of Copenhagen (514-1114-25-3000), Aarhus University (2022-0367531), and the University of Southern Denmark (12.250). All participants provided written informed consent for primary data collection, which also permitted secondary analyses without additional consent. Participation was voluntary and uncompensated, and all qualitative data were anonymized to ensure privacy and confidentiality. No identifiable images of participants are included in this paper or its supplementary materials.

## Results

### Development of the Logic Model and First Prototype

Below, we described the themes from interviews and discussions with stakeholders that served as the foundation for the logic model. In accordance with the MRC framework, logic models illustrate the program theory and how intervention activities can lead to the desired outcomes. This model served as a guide during the program's development, influencing both its content (psychoeducation, exercises, and examples), its design, and implementation based on contextual factors that could affect how users respond to the intervention. Finally, we describe how the first prototype was developed.

#### Summary of the Literature Review

Seven studies that tested self-guided interventions involving 648 participants (identified through EPDS, Beck Depression Inventory-Second Edition [BDI-II], or Patient Health Questionnaire-9 [PHQ-9] screenings) were reviewed. These interventions showed a significant reduction in depressive symptoms, but considerable heterogeneity among the studies limits their generalizability [10-12]. Notably, few studies incorporated formative research, including women with lived experience, even though codevelopment approaches can help tailor interventions to users’ needs, potentially resulting in more effective outcomes. This is the case of 2 trials evaluated MomMoodBooster, adapted from a group-based CBT program for PPD [11]. Development involved focus groups and usability testing (think-aloud method), leading to iterative refinements that improved engagement and functionality. The final program included psychoeducation, mood-monitoring, cognitive restructuring, communication skills, stress management, and baby care strategies. Similarly, Happy Mother (Pusan National University; The Catholic University of Korea) followed a multistage process emphasizing mobile app design [12]. Its CBT-based content was informed by literature reviews and maternal needs assessments, validated by experts, and tested for usability with mothers and health professionals. The app offered tools for psychoeducation, mood tracking, cognitive restructuring, positive activity engagement, and help-seeking. Despite showing promising results, most self-guided interventions remain outside routine maternal care. MomMoodBooster is an exception, demonstrating that integration at the clinic level is feasible [23]. However, evidence on scaling self-guided DMHIs across national maternal health care systems is lacking.

#### Themes From Round I Interviews, Group Discussions, and Focus Groups

Themes were identified and organized in four main categories: (1) need for a digital intervention based on experiences with current offers, (2) experiences of PPD, (3) preferences for program content and approach, and (4) design, technology, and implementation focused on engagement. Based on each theme, we made a recommendation for the program development that led to choices made in the development of iCARE (refer to Table S1 in Multimedia Appendix 4 for a full description of themes, quotes, and recommendations). To ensure anonymity, participants are labeled with “W” and a consecutive number (eg, W1 and W2), denoting women with lived experience of PPD.

##### Theme 1: Need for a Digital Intervention Based on Experiences With Current Offers

Across interviews and focus groups, participants emphasized the relevance of an internet-based intervention to fill gaps in maternal mental health care. Although all MHCPs encouraged women to contact their general practitioner when screening positive on the EPDS, 7 out of 20 municipalities were unable to offer any specific follow-up services, such as support groups or appointments with a municipal psychologist. MHCPs also highlighted the practical advantages of digital interventions, “They can save on transportation...it really matters in terms of time. You can do it when it suits you.” (Municipality 5, MHCP 1)

Across interviews and focus groups, participants emphasized the relevance of an internet-based intervention to fill gaps in maternal care. Mothers valued immediate support after screening or symptom onset, especially for those unsuited to group programs. They emphasized the convenience of digital intervention and highlighted its practicality as this participant described,

I think there are some pluses in having that app at hand. You don't have a lot of leeway, so it's difficult to get out of the house. It can be like a bridge out into the world… something that reflects what you feel inside, but also what is normal out in the world. And you have it at hand while you sit and breastfeed or feed the baby.

W2

MHCPs in resource-limited municipalities saw iCARE as especially relevant, noting that many mothers were already seeking advice online. They also highlighted that a digital format might help reduce stigma,

“And maybe also a bit less stigmatizing (while you are using the app) — you can fit it in.” (Municipality 5, MHCP 2)

At the same time, participants stressed that digital support should not replace in-person care. As one mother explained, “It can help improve things, but you still need some physical contact to get back on top” (W2). This feedback informed the inclusion of clear guidance within iCARE on how to access further treatment and professional support when needed, even though the program remained self-guided.

##### Theme 2: Experiences of PPD

Mothers described a wide range of PPD symptoms, including sadness, anxiety, irritability, and anger, and related them to experiences of stressors such as birth complications, breastfeeding challenges, infant health concerns, and social pressures. A recurrent pattern and feeling were guilt, shame, and misaligned expectations of motherhood, as this mother described, “I had this bad conscience towards not being able to be there and my expectation about being a mother.” (W1)

MHCPs and experts noted that societal ideals of “good motherhood” intensified anxiety and guilt when mothers’ experiences did not align with expectations. Relationships with partners, families, and health care providers were also mentioned as very important, either offering support or compounding distress. Most mothers described how their partners struggled to understand their emotional state:

It can be hard when you have a postpartum depression, and the father can find it extremely difficult to put himself in your shoes.

W9

Stigma remained a significant barrier to asking for help. One mother shared, “When I told people how bad I felt, they wondered if I could look after my child. That made me not want to talk.” (W2). Such reactions reinforced silence and suffering, contributing to prolonged distress. These findings informed iCARE’s emphasis on destigmatizing language, normalizing diverse postpartum experiences, and supporting mothers in articulating their needs within their relationships and wider social networks.

##### Theme 3: Preferences for Program Content and Approach

Participants wanted reliable, evidence-based information about PPD, its prevalence, and causes. Psychoeducation was considered essential not only for mothers but also for partners, as a better understanding could enable them to be more supportive. However, preferences for therapeutic approaches varied. Some preferred CBT strategies, “I think we used the cognitive diamond a lot in therapy, and that was actually what I could use the most” (W1), while others preferred metacognitive strategies, “...being aware that your thoughts are there, but you just have to choose to do something that doesn't respond to that thought” (W2). These differences highlighted the importance of offering flexible, multiapproach content, as one size does not fit all.

Beyond psychoeducation, participants requested practical coping tools and self-care strategies to manage guilt, unrealistic expectations, and anxiety. They stressed that recovery does not end with a short program as this mother described, “What should you do going forward (after the program) to take care of yourself?” (W8). This informed the inclusion of the last module to keep using the skills learned after the program.

Another recurring theme was the need for support in partner communication and shared responsibility. Mothers wanted tools that encourage constructive conversations about household responsibilities and parenting roles, reflecting a broader desire for interventions that address family dynamics, as this participant explained:

...(the program) can encourage conversations about division of tasks, practical things, making agreements about who is responsible for what at home, so it's not automatically the woman who keeps both baby and home.”

W2

In summary, participants envisioned a program combining psychoeducation, diverse therapeutic strategies, real-life narratives, audio-based content, coping exercises, and guidance for partner communication, ensuring iCARE is flexible and practical for everyday life.

##### Theme 4: Design, Technology, and Implementation for Increasing Engagement

Ease of navigation and clear initial framing were seen as essential for engagement. One mother noted, “One of the first things should be ‘How to get something out of this app,’ because time is scarce.” (W2) Flexibility to pause and resume at one’s own pace was highly valued, and participants recommended extending access beyond 2 months, given the recurrent nature of PPD.

Multimodal content was preferred, combining text, audio, video, and interactive exercises. Reducing text content was also mentioned by all. As 1 participant explained, “I want audio or video, not just reading” (W9). Graphics and exercises were expected to be simple, diverse, and adaptable to different contexts. They also emphasized the importance of easy-to-understand exercises offered in different modes that do not require much time. They noted that presenting exercises that can be done “on the move” could benefit some, such as “Audio files where the exercise comes in while you go for a walk and listen.” (W9)

Integration into maternal care was emphasized. MHCPs play a central role in screening and referral, and participants saw value in their involvement, “If the maternal health visitor presents it, she can follow up: ‘Have you looked at it?’” (W5).

This informed iCARE’s design for compatibility with universal PPD screening and home-visiting services.

#### Logic Model of iCARE

**Figure 2:**
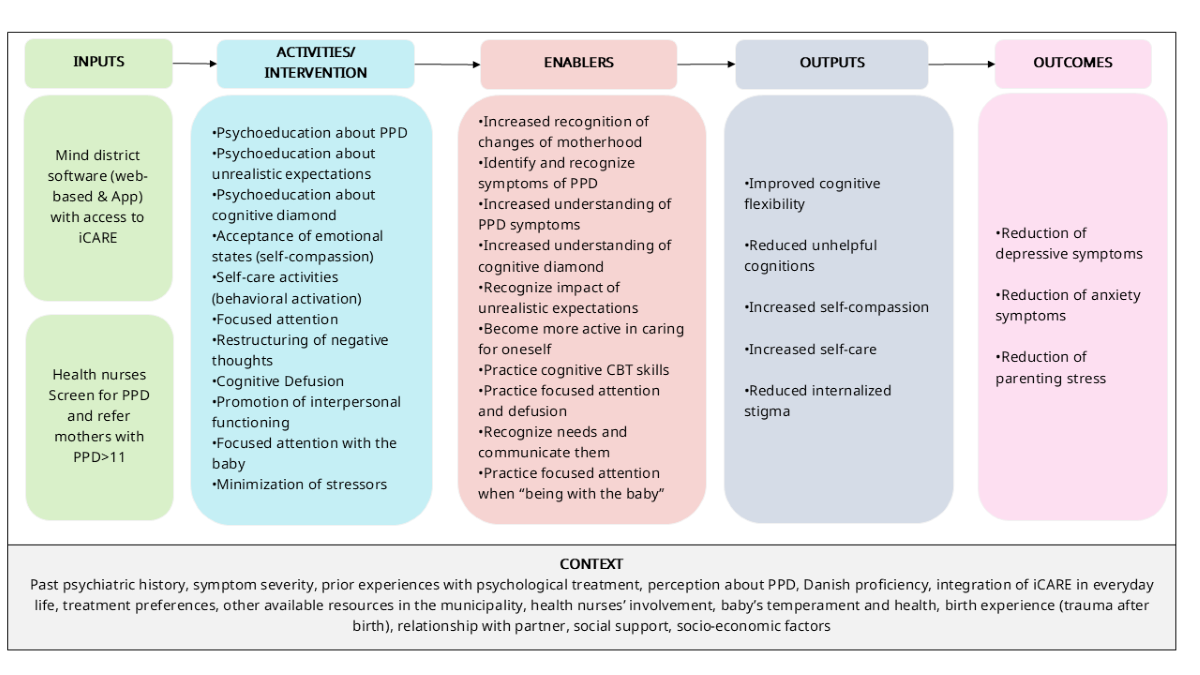
Logic model of iCARE. CBT: cognitive behavioral therapy; EPDS: Edinburgh Postnatal Depression Scale; PPD: postpartum depression.

We developed a logic model (Figure 2) based on the literature review and results from round one of interviews with mothers, MHCP, and mental health experts. Our logic model outlined the inputs and intervention components (activities), mechanisms of change (enablers), and both short-term and long-term outcomes, along with contextual factors that might impact the intervention's effectiveness. The logic model was an essential result for the development of the iCARE program prototype, as it outlined the expected mechanisms of the intervention to support mothers with symptoms of PPD and provided a framework that we further evaluated in the feasibility study and randomized control trial.

Theoretically, the program draws from CBT approaches to PPD, as they have been shown to be effective in digital interventions [24]. Accordingly, the intervention includes psychoeducation on postpartum depressive and anxiety symptoms, emphasizes behavioral activation through self-care activities, and helps participants identify the links between thoughts, feelings, bodily sensations, and actions. It also focuses on recognizing self-critical and negative thoughts, offering simple cognitive restructuring techniques to find alternative explanations and interpretations.

However, iCARE also incorporates ACT principles and therapeutic tools into the program content. Interviews with mothers revealed that women have different preferences regarding psychological approaches, and these preferences can influence their engagement with interventions. Additionally, guilt and self-criticism were found to be very common, a pattern observed in other studies [25]. One way to reduce self-criticism is to promote self-compassion, which ACT supports by encouraging acceptance of one’s feelings. In addition, recent evidence indicates that ACT is effective in preventing PPD and reducing anxiety symptoms [26]. While combining CBT and ACT might seem counterintuitive, CBT focuses on restructuring thoughts that lead to emotional distress, while ACT promotes accepting difficult thoughts and emotions using, for example, mindfulness techniques—this integration can be beneficial, as it offers more flexibility and a broader range of strategies [27]. Digital interventions, such as Be a Mom [28], which was developed to prevent PPD, already combine CBT and ACT approaches, showing both effectiveness and high retention rates. This suggests the potential value of integrating CBT and ACT principles and strategies in interventions for PPD [29].

#### Design and Content of First Prototype

Based on the results from the first round of data collection and the logic model, we developed the first prototype which we describe below.

### Structure, Content, and Functionality of the iCARE Program

The intervention consists of 7 modules covering different topics. Interviews with mothers and mental health experts revealed experiences that are unique to the postnatal period, which guided the tailoring of CBT and ACT components to mothers with PPD. Module 1 focuses on reflecting on normal changes associated with motherhood and psychoeducation about PPD, introduces self-care activities and finding focus. Module 2 introduces CBT principles and supports women in identifying how thoughts, feelings, actions, and bodily sensations are connected. Before introducing cognitive restructuring techniques, this module introduces simple diffusion techniques in the context of self-care activities. Module 3 introduces the role of positive emotions and how to refocus attention on them. It discusses how social pressures and unrealistic expectations about motherhood can affect thoughts about oneself. Then it introduces cognitive restructuring by focusing on how to find alternative responses to unrealistic expectations and self-criticism. Module 4 delves into additional examples of common difficult thoughts or interpretations and introduces cognitive restructuring techniques. Module 5 focuses on changes in relationships after giving birth, activating social networks, and promoting interpersonal functioning using assertive communication. Module 6 fosters a connection between mother and baby while coping with symptoms of PPD. Module 7 summarizes the skills learned and acknowledges future stressors and how to handle them. In addition, the intervention contains psychoeducation for partners about PPD, which mothers can choose to share through a PDF. Table S2 in Multimedia Appendix 5 describes in detail the components of each module.

Across all modules, the intervention encourages acceptance of emotional states as well as self-compassion. The language used is destigmatizing, depathologizing and nonjudgmental. Rather, the intervention addresses the user in an encouraging and inclusive manner normalizing the variety of experiences mothers may have. CBT techniques are also incorporated in Modules 6 and 7. For example, in Module 6, one example focuses on how self-criticism can prevent mothers from asking for support, and how to use some of the CBT techniques in response. In Module 7, one example focuses on how to use CBT techniques if the mother is having difficult thoughts when being with the baby.

Each module takes approximately 45 minutes to complete and must be followed in sequence. It is recommended to go through 1 module per week to leave time to reflect on the content and practice exercises. Pausing is possible at any point. The program integrates different learning modalities and seeks to be as interactive through various functionalities, including videos, text, exercises and activities, feedback loops, diaries, symptom checks, and audio files following the stories of 2 mothers who have experienced PPD. Mothers are accompanied by instructions, examples and encouragement throughout the progression of the program [29].

### Graphics and Representation

**Figure 3:**
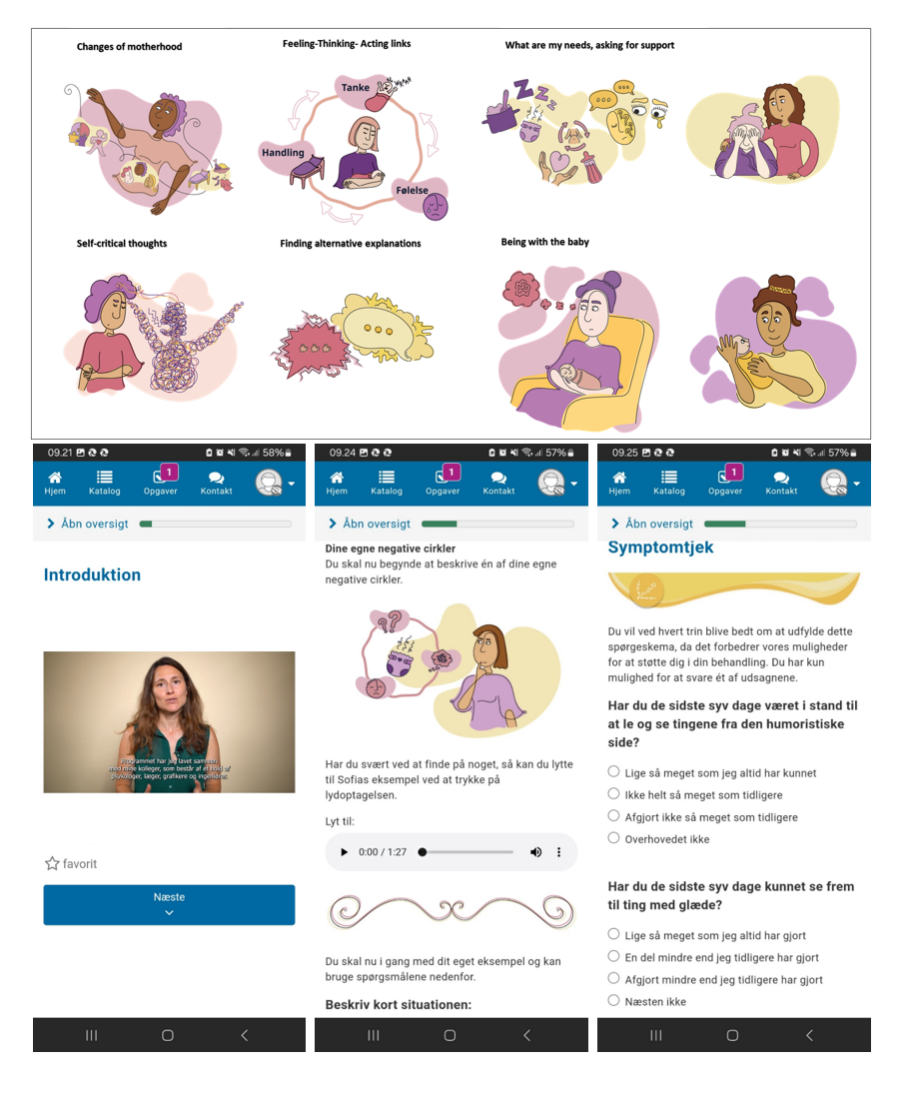
Examples of iCARE interface, graphics, and diverse representation. Explicit informed consent for the use of individual data or images in this manuscript was obtained from all participants.

Illustrations include mothers, partners, and babies with the intention of representing diverse experiences and setting the right mood for the intervention (Figure 3). Some illustrations move (GIF) and thereby engage the user in a different way. Each page is composed of illustrations and text in an appropriate ratio. In the videos, 2 of the research team’s psychologists present content with a focus on psychoeducation and teaching psychotherapeutic skills.

### Integration into Maternal Care

The intervention was designed to complement existing offers and could therefore be viewed as an offer without waiting time. We emphasize compatibility with current offers in the municipalities as MHCPs should not change existing practices and mothers can continue participating in group or in-person offers concurrently. iCARE seeks a clear referral pathway from screening. Based on feedback from MHCPs, training is provided for all MHCPs on referring mothers to the intervention and on using the EPDS. Even though some municipalities offer additional visits for mothers with EPDS scores above 7, we have chosen the cutoff score of 11 as the intervention was developed as indicated prevention for mothers that have elevated symptoms of depression and anxiety indicating a potential need for care. However, during the pilot study, we will explore whether this cutoff as an inclusion criterion is meaningful.

### Usability Test and Refinement of Prototype: Results From Interviews Round II

The usability test with the 5 mothers and the mental health experts provided positive feedback and agreement on the relevance of the content and the overall acceptability of iCARE. Design, illustrations, functionality, navigation, and clarity of instructions were also evaluated positively. At the same time, the analysis of feedback from prototype testing identified areas for refinement, which are presented below under major themes along with examples of changes made. A detailed summary of themes, quotes, and changes implemented and deferred can be found in Tables S3 and S4 in Multimedia Appendix 6.

#### Appropriateness of Content and Language

Participants valued the compassionate tone and nonpathologizing language. Mothers expressed appreciation for the approachable and supportive wording, describing that they “felt seen,” “not alone,” “mirrored,*”* and “hopeful.*”* These quotes illustrated what mothers thought about the program:

I actually think it feels accommodating, concrete and... Well, I don't know what to say. For me, it's just important not to be talked down to, I think, and I don't think you do that at all.

W5

There is language that is very different, and very different from symptom-focused, and visibilizing. It's a completely different language. I think it is one of the strengths of this.

W2

Feedback also addressed graphical presentation: 2 mothers recommended removing a pacifier icon and adjusting color palettes to make the design feel less gendered. The pacifier was removed, and color adjustments were made. Emotional safety was emphasized, with agreement that individuals with severe symptoms would need in-person support. Clarity about the program’s scope and referral pathways was strengthened.

Feedback about mothers’ stories was mixed: some participants appreciated hearing other women’s experiences, while others preferred not to listen to them. Audio examples were kept because they can be skipped.

Despite including a wide range of symptoms, some participants felt there was too much focus on sadness and self-criticism, and that symptoms such as irritability and anger were underrepresented. To address this, more diverse symptoms were introduced, and illustrations were updated. Experts recommended toning down diagnostic language and focusing more on distress and emotions to reduce stigma. Language was softened throughout, although references to PPD were retained in videos, which were not changed at this stage.

#### Motivation and Engagement

Participants highlighted that engaging with digital content can be challenging when experiencing postpartum symptoms. Audio content was highly valued for enabling multitasking, especially during baby feeding. Positive experience exercises were described as helpful, but cognitive restructuring felt demanding; participants requested step-by-step guidance and reassurance that pausing was possible. Experts recommend adding interactive elements and microfeedback to sustain engagement.

To address these points, we reframed the introduction to present iCARE as a “toolbox,” allowing mothers to use the tools most relevant to them. Statements and reminders were added to encourage effort and practice, and interactive features such as tick-box prompts and multiple-choice questions were introduced. Two additional audio files were added with mindfulness exercises, and instructions for browser read-aloud functionality were provided.

#### Inclusivity and Gender Representation

Language was revised to emphasize shared parental responsibility, replacing gendered terms such as “moms and dads” with “parents” as suggested by some mothers. Suggestions for more inclusive illustrations of family compositions were noted for future updates.

#### Clarity of Instructions and Data Use

Instructions were generally considered clear, but participants suggested making certain aspects more explicit. This included information about program access, monitoring, and who could see written responses. Two participants assumed their entries might be monitored, which indicated the need for repeated messaging that only EPDS responses were reviewed and only by the research team, and that emergency contacts should be used if needed. This was addressed by adding explicit statements about nonmonitoring of text and repeating emergency contacts. Reassurance text such as “okay to skip or return later” was also added.

#### Understanding of Therapeutic Method

Experts highlighted the importance of explaining the therapeutic approaches underpinning iCARE. One expert suggested clarifying the rationale for sequential steps and considering optional modules for flexibility. While introducing optional modules was not feasible at this stage, we reviewed examples to make sure they also reflect experiences of anxiety. An explanatory text was added to improve understanding of the therapeutic approach.

#### Usability, Layout, and Navigation

Overall, participants found the program easy to navigate but suggested refinements to reduce cognitive load and improve accessibility. Recommendations included collapsing sections, colocating illustrations with text, and splitting long introductions into shorter screens. Subtitles were added to videos to improve readability. Mobile optimization was affirmed as better than desktop, and layout adjustments were made for balance between graphics and text. Improvements to video performance and section collapsing were deferred to the next phase.

#### Summary of Changes

Most refinements involved text revisions aimed at enhancing clarity and reducing text, and minor graphic adjustments. Videos were not changed due to time constraints but will be reviewed during the feasibility study. Only subtitles were added to all videos. Repetition between video and text was minimized, and new audio examples were deferred. Future evaluations will assess whether the term “postpartum depression” feels pathologizing. We deferred developing new modules (eg, a module for mothers experiencing anxiety) and kept the modules sequential, instead of thematic and open from the beginning. This will be explored in the next phase.

## Discussion

### Principal Results

This study describes the formative development of iCARE, a self-guided digital intervention designed to complement Denmark’s maternal care system. Stakeholder input confirmed the need for timely, accessible mental health support following PPD screening and shaped multimodal content, compassionate language, and clear referral pathways. A distinctive feature of iCARE is its integration of CBT and ACT strategies, offering a flexible “toolbox” to accommodate diverse psychological preferences.

Usability testing of the prototype indicated a high acceptability, with participants valuing relatable narratives and audio features that fit into daily routines. Feedback led to refinements such as clearer instructions, interactive prompts, and additional mindfulness exercises. However, several challenges emerged. Cognitive restructuring exercises were perceived as demanding, and some mothers expressed concerns about the program’s sequential structure, which may have limited flexibility for those with fluctuating symptoms or different preferences. Engagement remains a critical risk for self-guided interventions, and sustaining motivation over time will require further testing.

Embedding iCARE within Denmark’s stepped care model, with MHCPs introducing the program during routine screening, was viewed as essential for uptake. Future research will evaluate the feasibility, effectiveness, and strategies to address engagement and retention, which are common barriers in DMHIs.

### Comparison With Prior Work

A recent bottom-up review of the lived experiences of PPD among women, co-designed and co-conducted by both experts by experience and academics [25], highlighted the pervasive guilt that many women with PPD experience, which is further compounded by societal norms around motherhood. Feelings of guilt and shame are indeed common reactions postpartum as recently documented in a Danish setting [30]. Prior qualitative studies have also emphasized how the differences and complexities women face during the perinatal period merit modifications to existing treatments such as CBT [31]. As we have also observed in our formative research, the findings emphasize the importance of incorporating the varied and nuanced experiences of mothers with PPD into practice and research. In this context, our findings advocate for advancing the debate on how digital interventions can better reflect and address the diverse realities of mothers experiencing PPD symptoms and help reduce guilt and stigma. One way to achieve this is by actively engaging women with different lived experiences throughout the development and evaluation of digital interventions.

Our development approach aligns with a few prior studies that have engaged with women with experiences of PPD in the development phase. Like them, we prioritized user-centered design, beginning with interviews and usability testing and incorporating CBT-based exercises such as psychoeducation, mood-monitoring, and self-care based on the literature review. However, our approach also diverges in important areas. Unlike similar digital self-guided interventions, MomMoodBooster and Happy Mother, we focused on integrating our self-guided digital intervention into existing maternal health care services [11,12]. This distinction led us to engage MHCPs early on, exploring practical pathways for implementation. Additionally, while both prior interventions were grounded in CBT, our study expanded the therapeutic framework by incorporating ACT tools, providing mothers with a more versatile “toolbox” of skills to manage symptoms of PPD. This approach has been proposed by other preventive interventions such as Be a Mom [32,33] and warrants further exploration. By comparing these development pathways, we highlight the evolving landscape of digital interventions for maternal mental health. While previous research underscores the value of formative work, our study aims to further explore how system integration and a blended therapeutic approach could enhance engagement.

### Limitations and Next Steps

While the study makes a strong case for iCARE’s potential, it acknowledges limitations in participant diversity. Despite we aimed for diversity, all mothers who participated in the study had at least high school education, Danish background, had experienced moderate to severe PPD symptoms and received in-person therapy, which could affect the generalizability of findings. Future research should examine how women with milder symptoms respond to the intervention’s content and language.

In addition, iCARE uses a fixed, sequential structure, requiring users to progress through modules step by step. Although this design promotes clarity and consistency, its rigidity may not fully accommodate the varied or fluctuating symptoms of mothers with PPD. Greater flexibility in pacing or content selection could enhance responsiveness.

In the next iteration, we aim to engage a more diverse group in terms of sociocultural background and symptom severity to refine iCARE’s content. Combined with pilot findings, this will inform further adaptation. Upcoming steps include evaluating the feasibility in a pilot study, its effectiveness, and retention in a randomized controlled trial. Given concerns about dropout rates in digital mental health interventions, strategies to sustain user engagement will be crucial. Furthermore, the integration of iCARE into Denmark’s stepped care model will offer an opportunity to test its scalability and alignment with existing maternal health care practices.

### Conclusions

Engaging with relevant stakeholders is essential for the development of an intervention that can meet the needs of the target population. In our study, mothers, MHCPs, and mental health experts allowed us to identify a range of pertinent aspects to consider in the development of iCARE and its refinement. The initial usability test suggests that the content of iCARE could be acceptable and appropriate for mothers with moderate symptoms of PPD.

Retention remains a critical challenge for DMHIs. The study’s iterative development process directly addressed this issue by involving end users in the design and refinement of iCARE. Features such as flexible pacing, relatable content, and multimodal delivery were tailored to sustain engagement. Following the MRC framework, we will continue tailoring the program further to accommodate diverse needs and explore its scalability within Denmark’s health care system during the pilot phase.

## Appendix

Multimedia Appendix 1 Interview guide with mothers.

Multimedia Appendix 2 Prototype testing interviews.

Multimedia Appendix 3 Interview guide with maternal health care professionals.

Multimedia Appendix 4 From themes to actions.

Multimedia Appendix 5 Summary of iCARE content.

Multimedia Appendix 6 Results from prototype testing.

## Abbreviations

ACT: Acceptance and Commitment Therapy
BDI-II: Beck Depression Inventory–Second Edition
CBT: cognitive behavioral therapy
DMHI: digital mental health intervention
EPDS: Edinburgh Postnatal Depression Scale
MHCP: maternal health care provider
MRC: Medical Research Council
PHQ-9: Patient Health Questionnaire-9
PPD: postpartum depression
RQA: rapid qualitative analysis

## Data Availability

The datasets generated or analyzed during this study are not publicly available due to the sensitive nature of the qualitative data, which consists of interview transcripts containing personal information. However, a summary of anonymized quotes is available from the corresponding author upon reasonable request.
