# Supplementary material for: iCARE Self-Guided Digital Intervention for Postpartum Depression in Danish Mothers: Formative Research Using User-Centered Design
Source: JMIR Form Res. 2026 May 13;10:e73948. doi: 10.2196/73948 (PMC13216761; doi:10.2196/73948)
Supplement: Multimedia Appendix 1 [file formative_v10i1e73948_app1.docx]

**Appendix 1_First Interview with mothers (translated from Danish)**

**Purpose**

Thank you for taking the time to meet with us and for agreeing to participate in this project. As we described over the phone, we are developing an internet-based intervention for women who have symptoms of depression and anxiety after childbirth.

We want to create an intervention that best meets the needs of these women by speaking with women who have previously experienced postpartum depression. Therefore, we would like to hear about your experiences and ask about the support you received, as well as what helped you feel better.

In addition to that, we would like to tell you a little more about internet-based interventions and hear your opinion on them. This will help us as we develop the various components of the intervention. Once we have a prototype of the intervention ready this summer, we would like to show it to you and get your feedback.

**Consent**

Go through the information sheet and consent form (see information sheet).

Remember to mention: anonymity and confidentiality / you can stop the interview at any time / withdraw consent at any time.

Remember to ask for permission to record the interview.

Do you have any questions before we begin?

**Background of the Informant**

Could you briefly introduce yourself and your background for being here today?
How old are you?
How many children do you have?
When did you experience postpartum depression? (Which pregnancy?)

**Experiences with Postpartum Depression**

Can you tell me about your experience with postpartum depression? How did it start?
How did you feel?
Were there any activities or routines that were difficult for you to do? Can you tell me more about that?
What kind of thoughts did you have about yourself?
Can you tell me what was the hardest part for you?

**Seeking Support**

When did you become aware of your postpartum depression and when did you decide to seek help?
Had you heard about postpartum depression before you began experiencing symptoms?

I would like to hear a little more about the support you received and how your experience with it was.
What kind of support did you receive?
How did you look for support?
What role did your health visitor play in discussions about postpartum depression and in helping you get support?

How did this support help you feel better?
You mentioned earlier that you felt (*) and thought (*); what helped you feel better? When did you start to feel better?
What did you learn about postpartum depression through this process and the support you received?
 Was there anything that didn’t work for you?
What changes did you make to cope with your depression?
Was there a type of support you wish you had received earlier?
Did you share your thoughts and feelings with people in your network (family, friends)? How was that?
What was your experience talking about it with others?
Did you feel heard/understood? Why?
Did you experience any stigma? Why?

Did you ever search for information or resources online during your postpartum depression? (If they say yes: Do you think the information was useful?)
What kind of information did you search for?
How did you use that information?

**Transition to Questions About the Intervention**

Thank you for sharing your experiences with postpartum depression and support.

Briefly summarize what was the greatest help for her in overcoming depression.

Now I would like to tell you a little more about the intervention we are developing, but first:

Have you heard of internet-based interventions before? Is it something you have used before or have previous experience with?

Ask after introducing the internet-based intervention.

What is your first impression? What do you think about it?
How do you think such an intervention would be helpful?

DESCRIPTION OF THE INTERVENTION**:** In internet-based interventions, you follow an online program at your own pace. Throughout the program, you are introduced to information that will help you gain a better understanding of what depression is. Additionally, you do various exercises to help manage and reduce your symptoms.

The program is based on the idea that your thoughts, actions, and feelings are interconnected. For example, when we are depressed, we typically have more negative thoughts (ADD EXAMPLE). We think more negatively about our experiences. Imagine that we put on a pair of dark sunglasses, which affects how we feel and act.

The intervention is self-guided and aims to give women more knowledge about postpartum depression as well as practical exercises that can reduce symptoms and help them feel better.

**Internet-based interventions: Program-related factors**

As I mentioned, there is no other person on the other side of the screen. What do you think could motivate women with postpartum depression to use such an intervention?

What kind of information would you want the intervention to contain?

How do you think it would be helpful for women experiencing postpartum depression symptoms?
What do you think is the most important thing for us to keep in mind when creating an internet-based treatment program?
Which (three) elements do you think would be most important for you, and why?

**Internet-based interventions: User-related factors**

How do you think an internet-based treatment would have helped you? (If they say it wouldn’t have helped: ask why and what would make it effective?)

Can you think of any potential challenges with using an internet-based intervention?
How would it fit into daily life with a newborn?

Time per session? (how long it should take)
Frequency?
To what extent do you think you would be able to complete the intervention? For example, if there are 6-8 modules stretching over 8 weeks, with each one lasting 30-45 minutes.

**Internet-based interventions: Technology-related factors**

Are there any specific features that you would like the intervention to include?

Show the graphics: These are some of the graphics we will include in the intervention. What is your first impression? What do you think about them?
Name three positive things about the graphics
Name three missing aspects of the graphics
For example, what do you think about videos of other women sharing their experiences? (If they think it's a good idea: do you prefer the setting/background to be more homey rather than clinical? For example, do you prefer the woman in the video to be sitting on a chair, should there be plants in the background, etc.?)
What do you think about small podcasts that you can listen to while walking?
What do you think about exercises (like homework) that you need to complete during the week? For example, keeping a journal where you can set weekly goals or identify your negative thoughts and learn to focus on positive thoughts instead?
Do you think the colors fit for an internet-based treatment aimed at postpartum depression? Why or why not?

**Conclusion**

We are now nearing the end. Are there any topics or points that we haven’t discussed that you expected we would touch on?
Briefly summarize (over the general points)

Thank you so much for your time and for sharing your story with us. Once we have developed the intervention, we would like to share it with you before we introduce it to other women. Would it be okay if we contact you again in that regard?
