# Supplementary material for: iCARE Self-Guided Digital Intervention for Postpartum Depression in Danish Mothers: Formative Research Using User-Centered Design
Source: JMIR Form Res. 2026 May 13;10:e73948. doi: 10.2196/73948 (PMC13216761; doi:10.2196/73948)
Supplement: Multimedia Appendix 2 [file formative_v10i1e73948_app2.docx]

**Appendix 2 – Protocol - iCARE user test of mothers with lived experience of postpartum depression (translated from Danish)**

1: Think aloud online interview introducing iCARE and module 1

2: Questions for each module answered on through SurveyXact

3: Online interview on overall experience with iCARE

**1: Think aloud online interview of mothers with lived experience of postpartum depression introducing iCARE and module 1**

| Presentation | present yourself |
| --- | --- |
| Introduction | Earlier this year, we interviewed you in connection with the research project on Internet-based treatment for postpartum depression ( iCARE ). This is a digital self-help intervention that works on the computer and on the phone. This intervention is for women with mild to moderate symptoms of postpartum depression, as severe symptoms require individualized therapy. It is important to emphasize that the intervention in all cases does not replace help from your own doctor or psychologist, or other offers such as mothers' groups .  Today we will present you with the prototype of the program and interview you to hear your opinion and feedback about iCARE . Thank you for taking the time to participate. |
| Purpose | As described on the phone, we will do a user test with you. The purpose is to test the program's functionality, usability and content. By testing the program with you and other participants, we will identify areas of improvement that will benefit future users of the intervention. Your feedback is therefore important.  Today we will do an online "think aloud" interview in relation to the introduction and the first step of the iCARE programme. Next, you have time to go through steps 2-7 on your own. You will receive a few questions for each step in advance, which will be answered through SurveyXact . Finally, we will conduct an interview with you about your overall experience of the program . How we structure the user test depends entirely on your time. If you only have time for some of these steps, that's fine. |
| This is how the test works | The iCARE program is so far only used for the user test and therefore cannot be considered as treatment. When we go through the program together and when you go through it alone, you therefore do not have to answer honestly nor do all the exercises, but of course you are welcome. We just ask you to imagine what it will feel like to go through the program.  What we will do today is called a think-aloud exercise. We will go through the first steps of the program and ask you to think out loud throughout. It may seem a little artificial, but try to respond out loud to the information and exercises you will be introduced to. We ask that you speak clearly and tell us what you see and do in the program. Try to describe what you think about the content, design, structure and interaction in the program.  You are encouraged to complete the test with as little assistance as possible. But you are of course welcome to ask questions along the way and we will also ask you questions. We will record the exercise and ask you to share your screen during the interview.  Since you have already given consent, you have received a link to the training platform. |
| Consent | - Review information sheet and consent form (if not already done) - When the user test is over, your user account will be closed, your e-mail address will be deleted and also your answers. - Remember to mention: anonymity and confidentiality / you have the option to interrupt the interview at any time / withdraw the consent form. - Remember to ask for permission to record the interview.   We record you on camera and sound. This data is only used internally and is only seen by me and my colleagues. We store your data securely for a maximum of one year. You can read more on the declaration of consent.   - Do you have any questions before we begin? |
| Important to remember | Before we get started, there are some important things to keep in mind when completing the tasks:   - It is called a user test and it is important that you know that we are NOT testing you and how good you are at using the program. We test the program whether the functions are easy enough for the user to use and whether the content is relevant. - You can do no wrong. - If you encounter problems along the way, you should know that it is not your fault, but the program's fault and the more errors/shortcomings you encounter, the more we can change and improve. - Be as honest as possible. If there is something in the program that you don't like or that seems confusing, say so! We are not picky, but interested in all kinds of feedback. |
| User profile and screen sharing | Have you created a profile and are you logged in to the training platform?  Want to share your screen with us? |
| Cognitive walk-through: | - What do you think of the first introduction video? - Is the video presentation of the program clear? - What is your first impression of the introduction to the program and overview of modules? - What do you think of the audio files with stories from mothers? - Are the technical and navigation instructions clear? - How would you experience going through a symptom check after each module? - What do you think of this introduction overall? - Introducing module 1, how do you find the changes with pregnancy and birth section and learning about postpartum depression? - Are the exercises on self-care and notice and refocus understandable and relevant to you? Do you need more guidance? - What do you think of module 1 overall? |
| General questions | - How do you experience the program? - How do you think the program works overall? - How do you feel about the structure, navigation, functionality and usability? - What do you think of the instructions along the way? - What do you think about the language used? - What kind of treatment do you think iCARE contains? - What do you think of the symptom checks? - What have you learned from the program? - How do you relate to the examples of Maria and Sofia? - What do you think of the videos and the music in them? - What is your overall attitude/perception of the illustrations? |

**2: Questions for each module answered on through SurveyXact for mothers with lived experience of postpartum depression**

The following questions were asked for each of the modules, questions 1-4 were answered by ticking one of the following options: Strongly agree, agree, neutral, disagree, strongly disagree.

1. This step was helpful.

- Comments (why or why was not this step was helpful)

1. This step was easy to understand and navigate.

- Comments (why or why was not this step was easy to understand and navigate)

1. 3. This step's exercises were understandable.

- Comments (why or why not the exercises were understandable)

1. This step's exercises made sense to me.

- Comments (why or why not the exercises made sense)

1. Do you have any other comments or feedback on this step (for example, what you liked or disliked)?

**3: Online interview of mothers with lived experience of postpartum depression on overall experience with iCARE**

| Introduction | Welcome to our second interview. You have now participated in a "think aloud" interview and reviewed the iCARE program for yourself. We invite you to this final interview to hear about your overall experience of the program. |
| --- | --- |
| Purpose | The purpose of this interview is to hear about your experience with the iCARE program in relation to all the aspects they want to share with us. |
| This is how the interview takes place | I want to ask you about a number of elements of the intervention and if there is something you do not remember, we can come back to it. I ask you open-ended questions and it is of course completely up to you what you want to answer. |
| Consent | - Remember to mention: anonymity and confidentiality / you have the option to interrupt the interview at any time / withdraw the consent form. - Remember to ask for permission to record the interview.   We record you on camera and sound. This data is only used internally and is only seen by me and my colleagues. We store your data securely for a maximum of one year. You can read more on the declaration of consent .   - Do you have any questions before we begin? |
| Important to remember | Once again, we encourage you to be as honest as possible. If there is something in the program that you don't like or that seems confusing, say so! We are not picky, but interested in all kinds of feedback. |
| **Structure and functionality** | - How did you think the iCARE program worked overall? - How did you find the structure, navigation, functionality, and usability? - What did you think of the instructions along the way? - What functionalities did you miss? - What did you find difficult in the program? - Did you experience any errors or elements that did not work? - How was the navigation to the diary and catalogue? - How long did it take you to learn how to use the program? - How long did it take you to complete a step? - Was it clear to you how far you had progressed in the program along the way? |
| **Design and multimodality** | - What was your overall attitude/perception of the illustrations? - How did you feel about the connection between the illustrations and the text? - How did you feel about the amount of text, illustrations, tasks, videos, audio clips, and music? - What did you think of the videos and the music in them? - Was there anything that bothered your eye? |
| **Content** | - What did you think of the program's processing method? - How was it to answer the questions with answer options? - What was it like to do the exercises? Were there any challenges (e.g., step 4 on cognitive restructuring was quite long)? - Was it clear that you could write as much or as little as you wanted? Or skip tasks? Not have to answer everything? - What did you think about the possibility to write in the diary? - What did you think about the content of the videos? - How did you feel about the stories (audio clips) with Sofia and Maria? (Were they relevant or relatable?) - How did you feel about going through the symptom checks after each step? - What did you think about the complexity of the content? (e.g., step 6 on how babies communicate + the exercise therein) - What did you think about the language used? - Was there anything in the program that you found stigmatizing (symptoms are no one's fault)? Anything in the language? - What had you learned through iCARE? |
| **Overall impression** | - How did you experience the iCARE program as a whole? - How did you think the structure, design, and content fit together? - What was missing in the program? |
| Additional feedback | - What do you think are the advantages or disadvantages of the program? - Is there anything in the program that has made you quit? - Is there anything we haven't asked that you would like to share with us? - Is there any other feedback you would like to give us? - Do you have any questions? - Can we send you updates or consult you on changes we make? |
