# Supplementary material for: iCARE Self-Guided Digital Intervention for Postpartum Depression in Danish Mothers: Formative Research Using User-Centered Design
Source: JMIR Form Res. 2026 May 13;10:e73948. doi: 10.2196/73948 (PMC13216761; doi:10.2196/73948)
Supplement: Multimedia Appendix 3 [file formative_v10i1e73948_app3.docx]

**Appendix 3_Focus group maternal health care providers (translated from Danish)**

**Semi-Structured Interview Guide – Health Visitors**

**Content:**

- Current practices of step care for PPD
- Priorities and expectations about iPPD (content and technology)
- Potential barriers and facilitators to engagement in internet-based therapy
- Expected role of health nurses in supporting implementation of iPPD

**Purpose:** Identify, characterize, and explain key mechanisms that promote and inhibit the implementation, embedding, and integration of new health technologies (iPPD) using Normalization Process Theory (NPT).

NPT proposes four constructs that represent different kinds of work that people do around implementing a new practice: Coherence, Cognitive Participation, Collective Action, and Reflexive Monitoring.

**Introduction / Purpose / Background**

Thank you for taking the time to meet with us today. As you know, we are developing an internet-based intervention for women with mild to moderate postpartum depression (PPD) symptoms. We have started working on developing the intervention, which will be ready in a year. We are meeting with women who have experienced postpartum depression. Now we would like to learn more about your experiences in screening women for PPD and providing support.

Since the project is still ongoing, our questions will focus more on your expectations, your understanding of your role, and your previous experiences with technology (e.g., using or recommending apps, websites, etc.). For an implementation to be sustainable and accepted, it also requires that you, as close collaborators, evaluate it as a useful program. Your perspective on such digital implementations is therefore very important. We would also like to thank you for agreeing to participate in this interview and for sharing your experiences, knowledge, and input with us.

**Consent** Go through:

- Consent statement
- Anonymity and confidentiality
- Right to withdraw consent
- Permission to record audio

**Informants' Background** (their profession, specialization, etc.) Can you briefly introduce yourself?

- What is your role?
- How many years have you worked as a health visitor?

**A Normal Workday / Current Practices Regarding Postpartum Depression**

First, we would like to learn about your role in screening and supporting women with postpartum depression.

Can you tell us how you screen women for postpartum depression? (Adjust questions based on responses)

- How do you use the EPDS?
- How do you introduce it?
- Have you experienced women refusing to be screened? What would you do in such a situation?

What criteria do you use to assess whether a woman is at risk for postpartum depression?

- If women are not screened with the EPDS, how do you assess their mental health?

What are the reasons for a woman not being screened for postpartum depression? For example, are there women who refuse to be screened?

**What They See as Their Existing Role in Supporting Women's Mental Health**

What do you do when a woman shows symptoms of postpartum depression?

- If you offer an additional visit, what do you do during such visits?
- If you offer a mother’s group, what happens in those groups? Who leads them? Is there a waiting list or ongoing enrollment?
- Do you use any digital resources in your work with mothers? (e.g., website, videos, social media?)
  Do you experience any challenges in providing support to women with postpartum depression? Can you give me an example? (Lack of knowledge, taboo, no resources to refer women to)

**Views on PPD**

Based on your experience, why do you think women experience postpartum depression? What symptoms do women often experience?

**Previous Experience with Digital Solutions/Technology**

Have you had experience using technology or digital solutions in your work (other online interventions, apps, videos, other digital resources)?

- What did you have to change in your work when you started using technology?
  Did you change anything in the technology to make it work better for you or the women?
  Can you give a concrete example of a technological solution in your work that has been successful for you?
- Why do you think it was successful?
  Can you think of a specific example where it didn’t work?
- Why do you think it didn’t work?

**Questions about iCARE**

DESCRIBE THE INTERVENTION:

Now we would like to ask you some questions to help us implement the intervention in the best way.
In internet-based interventions, women follow an online program at their own pace. During the program, they will be introduced to information that helps them understand what depression is. Additionally, they will complete various exercises to help them manage their symptoms and reduce them.

The program is based on the idea that their thoughts, actions, and feelings are interconnected. The intervention is self-guided and aims to provide women with more knowledge about postpartum depression as well as practical exercises to reduce symptoms and help them feel better.

The intervention will consist of 6 to 8 sessions, each lasting 45 minutes. Women will have up to 8 weeks to complete them.

**Motivation and Expectations from Health Visitors**

What is your motivation for participating in this project?
What do you expect from iPPD?
Can you imagine that there will be other types of tasks and responsibilities for you once iPPD is implemented?

- IT-related questions/tasks.
- Do you have any concerns related to that?
- How do you think it will affect your workflow?
- Do you think this new intervention will affect your role as a health visitor? How?

Have you thought about the content of this new digital intervention?

- What kind of support do women with PPD need?
- Do you have any suggestions for the content we should include?

In the Future, If the Intervention Works and We Offer It to Everyone, Do You Think Health Visitors Could Have a Role in Helping Women Engage with the Intervention? (e.g., reminders via phone calls?) Will It Impact Their Workload?

**Expectations for Women's Engagement**

How do you think women will react to such an offer?

- Do you see the benefits of offering PPD treatment digitally?
- Is there anything you believe could already be a problem in practice?

What kind of challenges could women experience when using iPPD?

**General Knowledge / Their Thoughts on the Project**

We have now gone through all my questions. If you have any additional thoughts or points, please feel free to share them.
Or if you feel a specific area hasn’t been covered, please let us know.

**Summary**

Again, thank you so much for your time and for sharing your thoughts and perspective on this project. I feel that I’ve definitely gathered some important and valuable insights that we can use in our ongoing work.
