# Supplementary material for: iCARE Self-Guided Digital Intervention for Postpartum Depression in Danish Mothers: Formative Research Using User-Centered Design
Source: JMIR Form Res. 2026 May 13;10:e73948. doi: 10.2196/73948 (PMC13216761; doi:10.2196/73948)
Supplement: Multimedia Appendix 4 [file formative_v10i1e73948_app4.docx]

**Appendix 4: From themes to action in the development of iCARE**

| **Table S1. From themes to action in the development of iCARE** | | | | |
| --- | --- | --- | --- | --- |
|  | **Themes** | **Recommendation for program development** | **Recommendations and action taken** | **Quotes** |
| **Need for a digital intervention** | | | | |
|  | Limited and unequal offers across municipalities | Do not offer iCARE to mothers with severe cases of postpartum depression, without ensuring immediate in person support.  Facilitate access to in-person services for all users beyond iCARE | - Intervention targets mild and moderate symptoms - Include a symptom checklist after each module to identify users with suicidal thoughts and severe symptoms (EPDS >18), follow up by phone to facilitate referral - Encourage referral if women do not experience progress - Offer intervention right after screening - Allow referral in the first 6 months after birth | Quote n/a |
|  | Digital is less stigmatizing |  |  | *And maybe also a bit less stigmatizing, because you can do it when the older kids are… You don’t need to involve the mother-in-law in all of this if you don’t want to, because you don’t need a babysitter — you can fit it in*. (municipality 5, MHCP 2) |
|  | Easily available |  |  | *So I think that there are some pluses in having that app at hand. In practical terms, you don't have a lot of leeway, so it's difficult to get out of the house, so it can be like this - I think it can be like a bridge out into the world into the more normal. So that there is something in that app that reflects what is there - both what you feel inside, but also what is normal out in the world. And that you have it close at hand while you sit and breastfeed or give a bottle. I think that there are some great advantages in that. Availability, ok? (W2)*  *But they save on transportation, most of them… I mean… I also think it really matters in terms of time… That they can choose to join or not… That you can do it at times that just suit you.* (municipality 5, MHCP 1) |
|  | Preference for individual support |  |  | *It didn't make much difference (group therapy). It was nice knowing others felt like me, but a psychologist could have told me that. It was insane listening to others' stories when I was just trying to survive. (W1)* |
|  | In person support necessary when experiencing severe symptoms |  |  | *I don't think you will be able to treat postpartum depression with it (internet-based intervention). I think you can help improve it, but I think you have to have some physical contact with someone to be able to get back to the top (W3)* |
| **Experiences of PPD** | | | | |
|  | Diversity in symptoms and onset | Recognize diversity of symptoms so that all users can feel recognized | - *Module 1:* Include a symptom list beyond the EPDS items that show diversity of emotional experiences and reactions where mothers can recognize themselves. | *Sounds, like loud sounds, his crying, slamming doors, ambulances and so on, it goes straight into my nervous system and then I get insanely angry, like really extremely angry. (W5)*  *I constantly felt that I was a bit of a burden, not towards her, but I just didn’t want to be a burden. W1* |
|  | Diversity of stressors linked to distress | Recognize the diversity of stressors that are link to distress and recognize how challenging these might be fostering empathy towards mothers’ experiences | - *Module 1:* recognize a wide range of stressors during the perinatal period. Video and text explain a wide range of stressors that can lead to symptoms of PPD. These include pregnancy and birth experiences, the baby’s temperament and health, social expectations. | *As soon as my physical pain (after complicated birth) and feeling unwell had settled after the three months, then it was like I collapsed mentally. A lot of things just came flooding in, because I had hardly been able to do anything in those three months. (W9)* |
|  | Internalized Unrealistic social expectations about motherhood | Recognize social pressures about motherhood and normalize different emotional experiences about motherhood. | *Module 3:* include a section on motherhood, recognize social pressures about motherhood and normalize different emotional experiences. Support women recognize how societies unrealistic expectations affect how they feel and think about themselves. | *I just really wanted to be that mother who managed it all and made mash from scratch and had energy for everything. - It was a lot that I regretted and a lot that I was probably not fit to be a mother. That's how I felt. That I wasn't competent enough for that at all, when I looked at how everyone else managed it. (W1)* |
|  | Pressure to breastfeed affects mothers’ mental health | Support mothers’ choices with baby feeding. Break stereotypes about mothers that do not breastfeed. | - *Examples*: Use an example of a mother that cannot breastfeed and uses the bottle. Normalize that some mothers can’t or don’t want to breastfeed. Promote self-compassion around expectations that are not met. - *Personal stories*: one of the mothers feeds the baby with a bottle. | *A screaming child who doesn't get enough food either, because my milk hasn't flowed properly either. So he is hungry. (W2)* |
|  | Illness and temperament of the child put extra pressure on the mother | Recognize links between infants’ temperament and parents’ emotional responses.  Provide strategies to regulate mothers’ emotions when infants are dysregulated | - In exercises, include examples about infants that are more sensitive or hard to calm down and how that can trigger more negative thoughts about oneself. - *Personal stories*: One of the mothers has a baby that is very hard to calm. - *Module 6*: section on baby’s states and how to be with them based on what state they are in. Section on managing thought and feelings when the baby is unsettled. Using CBT skills to respond to negative thoughts about one-self and practice self-compassion to acknowledge that helping the baby south can be challenging. | *He had colic for the first five months, and that in itself is quite challenging, I think that if it hadn't been like that, I might have healed something sooner." – (W5)* |
|  | Guilt and shame lead to more pain | Support mothers recognize what factors lead to guilt and shame and destigmatize symptoms of depression and anxiety | - Across all modules, promote self-compassion and acceptance of difficult feelings - Use destigmatize language - *Module 2-4*: Understand the interaction between thoughts, emotions and actions. Examples are meant to support women recognize factors that leads to guilt and shame - Strategies to break the shame and guilt circles (CBT skills, self-compassion) | *The shame of having thought that way or felt that way, and I actually think that it is the shame that has followed me since, what's the word, as the worst enemy into this whole post-partum reaction. And I have been able to hear that too, when I have talked to others who have also (…) had a post-partum reaction. (W7)* |
|  | Judgmental attitudes and opinions of relatives and friends impact mental health | Recognize the role of social relationships in PPD | - Module 5: Module on activating social support and assertive communication to express emotional and practical needs | *I felt that everyone had an opinion on how I did it and whether I did it well enough, I felt wildly judged every time. (W1)* |
|  | Relationship with partner under pressure | Provide information for partners and focus on improving communication. | - Include partner materials that mothers can access, download and share with their partners. - Materials include information to support understanding about PPD, how to be supportive for the partner that experiences PPD, and how to care for oneself. - *Module 5*: includes examples to express your needs to partners | *We pulled apart, we couldn't really, he could see that things were bad, but he had no idea what to do. He was as powerless and bewildered as I was...So somewhere I started to hate him a little bit, I felt that he was helping to make me sick* (W2) |
|  | Isolation | Reduce isolation by supporting mothers activate their social networks | - *Module 5:* section on activating social networks | *So, the transition from being alone with my husband to becoming a mother and what the hell does it mean to be a mother and the way society is structured - that you are alone. So, the thing about thinking “God, we have to deal with this alone”, [father's name], me and [child 1], is that it dawned on me, I do not believe that it is meant to be like this. I think that we should be many people around. As they say: “It takes a village to raise a child”.*  (W6) |
| **Preferences for program content** **and approach** | | | | |
|  | Different symptoms respond to different psychological approaches | Consider elements of other therapeutic approaches, in addition to cognitive behavioral therapy, that can be incorporated. | - Across the modules CBT serves to identify the emotion, cognition, behavior link. - Across the modules CBT serves to identify the effects of negative thoughts and unrealistic expectations on feelings and behavior. - Simple restructuring skills and exercises are presented to find alternative thoughts. - ACT skills: Focus attention exercise (based on mindfulness) to support self-care activities and being with the baby, diffusion exercise to respond to intrusive thoughts when doing self-care activities - Promote self-compassion by using non-judgmental language and acceptance of emotional reactions | *I think we used the cognitive diamond a lot in that group therapy program and that was actually what I could use the most.* (W1)  *So if you can draw some inspiration from the metacognitive that it's more about being aware that they're there and that it pops up, but that you just have to choose to do something that doesn't respond to that thought.* (W2) |
|  | Changes of motherhood | Recognize that motherhood brings changes that require adaptation for everyone | - *Module 1*: focus on how pregnancy and motherhood change body, mind, identity, relationships and recognize how these require an adaptation process. - *Module 3*: Debunk myths about motherhood that lead to unrealistic expectations. | *I think that maybe there are some almost universal topics, and the thing about being told something about it and getting some tools to navigate the loss of control or the feeling of inadequacy or the loss of your old life, which was certainly also a theme for me.* (W1) |
|  | Need for understanding PPD | General information about postpartum depression, its prevalence and clarify the severity of postpartum depression | - *Module 1*: Psychoeducation about PPD. | *Well, that would be all the basics, what are some symptoms you might experience, how many get it, and especially the thing about the partner risking going down with it… There is also something around, so what can you do, what the science says about what works.* (W5) |
|  | Support from family and community | Provide psychoeducation on the importance of social support and offer an exercise to recognize emotional and practical needs and communicate them. | - *Module 5: focus on identifying needs and communicate needs to others. How to activate your networks.* | *It cannot replace having a forum with other people who have either tried the same thing, but if you can somehow also in that app also be aware of how to build a bridge. That it helps women reach out. I think that is really important. That you get to know what options you actually have and then some good advice in relation to, for example, when all people say "Just let us know if we have to help with something", shut up, you heard it a lot. That's not good enough, that is, if you can tell in some way that you can ask for help, tell your mother that she should cook for you. So it is very specific what you can ask for help with. (W2)* |
|  | Support for partners | Include information for the partner about PPD, its potential effects on the relationship, how to support a person with PPD. | - *Pamphlet about PPD for partners: This includes psychoeducation about PPD, information about how PPD can also affect the non-birthing parent, how to support your partner when is experiencing PPD, and where to reach for help.* | *You have to have each other's back, because otherwise it just brings one even further out. So, so one thing is such a theme about the partnership, it could also be really nice if there were partner sessions. (*W8) |
|  | Improve communication with partner | Support assertive communication between mothers and their partners. | - *Module 5: include examples on assertive communication with partners* | *At least something that encourages conversations about some things, the division of tasks, practical things, making some agreements about who is responsible for what at home, so it's not automatically the woman who keeps both baby and home. (*W2) |
|  | Mirroring other women's stories | Include stories from other women who talk about their experience  Use these stories to show what can help understanding oneself and cope with symptoms of PPD | - *Audio clips: Follow the story of two women that experience symptoms of PPD in different ways. Include 1-2 short clips in each module presenting both their experiences, thoughts and feelings and how they managed and coped with them.* | *So sometimes it was really just us mothers who talked to each other, but it was also incredibly nice because as mothers with postpartum depression, you can just spar a lot together even though you are in different situations. (W9)*  *It would give you a bit of a feeling of being met in your own crazy thoughts (...) then you can think "well okay, so I'm not alone. (W5)* |
|  | How to care for yourself during and after the program | Support to recognize and cope with stressors  Include a session on what to do after the program finishes | - *Module 1* and across the program focus on self-care activities - *Module 7:* section on how to identify stressors and use the learned skills to cope with them - *Reminders about asking for a referral to a psychologist.* | *And then awareness that the physical part, that you get light and air, sleep, food, the basics, that it's simply not something you can skip...* (W2)  *And the same with the ending… And what should you do going forward to take care of yourself. Because you are not healthy just because you have gone through such a 6-8 week program* (W8) |
|  | Strategies to cope with self-blame | Provide strategies to cope and respond to self-critical thoughts |  | *I can have a week at a time when I just have a meltdown, but damn how frustrating it is. So, I feel like a super bad person, and a super bad mother, and a super bad partner…. How do I learn to deal with it in the first place.? (*W8) |
| **Design, Technology and implementation for increasing engagement** | | | | |
|  | Clear and easy presentation | Content presented in a clear way so that one can easily navigate the intervention.  Clarify the purpose of the intervention, e.g. in the form of a short introductory video. | - *Introductory video* | *You also talked about it being modules (...) then you can say ‘oh yes I was in that module and then I’ll just start from the beginning if I’ve forgotten it’ (...) Short and clearly delimited, so you know where you are in it*. (W5)  *It should be ‘This is how you get something out of this app’, that should be one of the first things you meet, because time is a really scarce resource when you’re a new parent.* (W2) |
|  | Flexibility to follow at your own pace with a clear structure | Contain technological features that enable flexibility in its use, e.g. possibility of a pause button. | - Each module is divided into short sections. After each section, there is a next button. This allows users to take breaks if needed. - Users can pause in the middle of a module and come back to where they left it. - Users can go back to modules they have done at any time - Users can save sections and exercises that they liked under Favorites | *If you can tap in and out of it.* (W5)  *That you can do it at your own pace..."how am I going to logistically make it work with this little child" (...) This (the intervention) is absolutely brilliant, I imagine that you can also go in and out of it or something.* ( W6) |
|  | Structure and timing | Suggest leaving few days between modules. Find time to follow intervention | - *Module 1:* present structure and 7 modules. Encourage mothers to set time a part to follow the intervention. | *It was important to be committed to and have a specific time every week for the sessions. So, my child wasn't there, and I had time off.* (W8) |
|  | Multiple learning modalities | Avoid text-heavy modules. Include audio and video content | - Each modules combines text, video, audios, exercises with text boxes and clicking exercises. | *So it's not just, uh, two A4 pages with symptoms, and then you can sit and read through them, but that you might get them plotted in some way in a small figure or something... so that it becomes more, well, that's also the symptoms I have-...(W8)*    *That it doesn't become too text-heavy...that you make a video that explains very well how to do the exercise (W8)* |
|  | Images and graphics that represent diversity of families and experiences |  | - Images include a range of skin colors and family configurations | *I think it looks nice. I see various parenting types and relationships, and there are mirrored feelings. These illustrations offer many possibilities, which I think is really good.* It’s always a delicate balance; the images shouldn’t be too cartoonish, but they should still be relatable (W8) |
|  | Avoid lengthy sessions | Each module should last less than 45 min. |  | *As long as it only lasts 45 minutes, I think it would be possible, at least in my family, to fit it into a weekend or an evening or something. I think if it goes much beyond 45 minutes, then it starts to get difficult... (*W1) |
|  | Personalize treatment | Have a range of modules that mothers follow based on their needs | - The text emphasizes the uniqueness of each mother and how some exercises and content may work better based on individual preferences. - Combine CBT with ACT and self-compassion - All mothers are encouraged to follow all 7 Modules - Possibility of developing extra modules after the pilot | *Maybe some boxes you can press into with different subjects, so if anxiety is related to your PPD, you could click on that section and get information about it. (W9)* |
|  | Encouragement from nurses | Health nurses should be largely involved to ensure successful implementation.  Resources should also be set aside for the training of health nurses in screening and referring. | - Nurses receive training to recruit and refer mothers to the intervention - Nurses receive a 2h training on screening of PPD (refresher) - Weekly open hotline for nurses to consult about referral of mothers to iCARE | *I had good support from my health nurse. She took care of me, asked good questions, and acknowledged my feelings.* (W9) |
|  | Immediate offer after screening | Consideration should be given to introducing iPPD early in the course of PPD as part of a stepped care approach. | - Referral after screening. | *I think if it's internet-based, you might as well be ahead of it.* (W9) |
