# Supplementary material for: iCARE Self-Guided Digital Intervention for Postpartum Depression in Danish Mothers: Formative Research Using User-Centered Design
Source: JMIR Form Res. 2026 May 13;10:e73948. doi: 10.2196/73948 (PMC13216761; doi:10.2196/73948)
Supplement: Multimedia Appendix 5 [file formative_v10i1e73948_app5.docx]

**Appendix 5**

| **Table S2. Summary of iCARE sessions** | | | | | |
| --- | --- | --- | --- | --- | --- |
| **Module** | **Main Content Themes** | **Main Diary/Weekly Activity** | **Audio clips with mothers’ stories** | **Videos** | **Symptom check** |
| **1. Understanding postpartum depression** | Psychoeducation; thought–emotion–behavior links; awareness of triggers; self-compassion; grounding practices | Daily self-care activity (and grounding exercice) | - | 5 | no |
| **2. Thoughts, emotions, and actions** | Cognitive diamond; negative cycles; emotions vs thoughts; unhooking from difficult thoughts | Identify a negative cycle; grounding/unhooking activity | 1 | 2 | yes |
| **3. Developing skills to manage negative thoughts I** | Monitoring daily positive experiences (behavioral activation); identifying unrealistic expectations about motherhood and self-critical thought; finding alternative responses to self-critical thoughts | Daily positive experience | 1 | 3 | yes |
| **4. Developing skills to manage negative thoughts II** | Identifying unhelpful interpretations; practicing cognitive restructuring; reviewing progress across modules | Challenging negative thoughts with restructuring | 2 | 2 | yes |
| **5. Communicating needs and asking for help** | Recognize changes in relationships; identifying needs; assertive communication and activate social support; using CBT skills when negative thoughts interfere with relationships | Identifying and communicating needs ("My network") | 2 | 1 | yes |
| **6. Being with your baby** | Understanding baby signals; recognizing thought–emotion–behavior cycles in parenting; managing thoughts/feelings while caregiving | Simple activities to connect with the baby | 2 | 4 | yes |
| **7. Putting skills together and reducing stressors** | Maintaining gains; identifying early signs; stressor management; reviewing helpful cognitive and behavioral tools | Write stressors, early signs, and action plan | 2 | 1 | yes |
