# Supplementary material for: iCARE Self-Guided Digital Intervention for Postpartum Depression in Danish Mothers: Formative Research Using User-Centered Design
Source: JMIR Form Res. 2026 May 13;10:e73948. doi: 10.2196/73948 (PMC13216761; doi:10.2196/73948)
Supplement: Multimedia Appendix 6 [file formative_v10i1e73948_app6.docx]

**Appendix 6. Results from prototype testing**

**Table S3** provides a summary of the feedback from prototype testing that was analyzed using rapid qualitative analysis.

**Table S4** provides a more detailed analysis of the data generated through prototype testing with women with lived experience of PPD. This analysis was done after the first round of reviews and confirms the analysis produced by the rapid qualitative analysis. It also provides quotes for the different themes and subthemes. The quotes were shortened for the sake of clarity

| **Table S3. Feedback from end-users and mental health experts through the development process and changes made to iCARE analyzed via rapid qualitative analysis** | | | | |
| --- | --- | --- | --- | --- |
| **Theme/End-user and expert feedback on intervention components** | **Changes made** | **Example - changed from** | **Example - changed to or added** | **No changes were made (will be tested during Pilot phase)** |
| **Appropriateness of content and language** | | | | |
| Appreciation of content on self-care, positive experiences every day, challenging negative thoughts, audios, interaction with the baby, support from network and partner guide | Positive feedback. No changes needed. |  |  |  |
| Exercises with the baby appreciated due to the concrete examples and emphasis on play | Positive feedback. No changes needed. |  |  |  |
| **Appropriateness of content: videos** | | | | |
| Text along the video of mom and baby could describe instances of rupture and repair | Recognize that as parents we might miss the babies signal and with time, we can learn what babies needs  Use the video of the mom and the baby to show an example of rupture and repair in the connection |  |  | *"In the video, the mother doesn't notice the baby's need for a break at first and keeps singing and cycling her legs. It often happens that we as parents miss a signal from the baby and this is completely normal.   Did you notice that after a while the mother realized that the baby needed a break and to be calmed down?"* |
| Avoid the emphasis on attachment | Use of bond instead of "attachment" | *"It's completely normal not to feel close to your baby in the first days or weeks after birth."* | *"It's perfectly normal not to feel great love or closeness with your baby or a strong emotional connection to your baby in the first days or weeks after birth."* |  |
| Second video could be warmer, could be a women speaking about symptoms (instead of a man and a women), simpler language, less about why symptoms arise. |  |  |  | No videos changed due to time. Will be revised during the pilot study with a larger sample |
| **Appropriateness of content: graphics** | | | | |
| Illustrations emotionally appropriate, diverse and representative and color tones and moving illustrations appreciated | Positive feedback. No changes are needed. |  |  |  |
| The pacifier in the transitions should be removed, it should be about mothers not babies. | Illustration of pacifier removed |  |  |  |
| **Appropriateness of content: destigmatizing language and diversity of symptoms** | | | | |
| Appreciation of compassionate non-stigmatizing language: approachability, for everyone, encompasses widely, addresses taboos and misconceptions, not pathologizing, patronizing or too emotional, feel seen, "a place for me", not being alone, mirroring, hope and | Positive feedback. No changes are needed. |  |  |  |
| More diverse symptoms should be represented | Changed to reflecting which symptoms depression may entail | *"The program is designed to help women who are at risk of developing postpartum depression or who are already experiencing symptoms of depression and anxiety."* | *"This treatment program is designed to help mothers who are at risk of developing postpartum depression as a preventative measure or who are already experiencing symptoms of depression including anxiety, irritability and sadness."* |  |
|  | Check list of symptoms expanded to include more symptoms |  | *"I am irritable, angry or aggressive."   "I feel like running away from my life."* |  |
|  | Including range of symptoms | *"You probably experience times when the depressive symptoms come back."* | *"You probably experience times when sadness, irritability and anxiety come back. This is completely normal."* |  |
| Less focus on thoughts being negative | Using difficult/self-critical thoughts over negative thoughts | *"With postpartum depression, you typically experience more negative thinking. This thinking is often automatic."* | *"When you become pregnant, you experience many changes. When you're adjusting to the new situation or experiencing fatigue, for example, it's normal to have thoughts like the ones you see below. "* |  |
| Tone down language of symptoms and depression and focus more on distress and emotions (treatment vs. prevention) | Language minimally revised | *"You probably experience times when the depressive symptoms come back."* | *"You probably experience times when sadness, irritability and anxiety come back. This is completely normal."* | Assess whether use of the term postpartum depression is well received by mothers, or it is felt as pathologizing |
| Recognize the reality of feeling guilt | Changed to acknowledge the existence of guilt instead of prescribing ‘do not feel guilty’ | *"So don't feel sad or guilty if you experience these things."* | *"This is important to remind yourself if you are struggling with guilt about how you think or feel about your child"* |  |
| Recognize that mothers may not at first like the way their babies look | Adjectives deleted in exercise to connect with the baby and talk to the baby | *"Describe what you see or do: "I see your little eyes, I see your beautiful face, I see you, I touch your little feet, I am home with you."* | *"Describe what you see or do: "I see your eyes, I see your face, I see you, I touch your little feet, I am at home with you."* |  |
| Recognize difficulty in assessing the baby's needs | Added that it is not always possible to ascertain the baby's needs | *"Sometimes it can be frustrating for you and your baby if you try to interact with them and they're not ready."* | *"Sometimes it can be frustrating for you and your baby if you try to interact with him/her and he/she is not ready. We don't always know what our baby needs are and that's ok. Just as we don't expect others to always know what we need, we can't expect ourselves to always know what our baby needs."* |  |
| Introduce more diversity in the audio examples of the two mothers |  |  |  | No audios changed due to time constrains. Revise audios to make them more diverse (for example, one mothers could be single or have a partner of the same gender) |
| **Motivation and engagement** | | | | |
| Include more options and optionality | Present iCARE as a toolbox in introduction module | *"The program is a bit like an online psychologist. You work through the same steps you would experience in regular therapy - at your own pace."* | *"The program is a bit like an online psychologist, but without sitting face-to-face with another person. You work with the same tools that you would experience in a regular treatment. The treatment can be considered a toolbox where you can work with the tools that speak to you the most and leave others out."* |  |
|  | Option to opt-out in in-site exercises |  | *" I would like to move on without replying "* |  |
|  | Give mothers examples in addition to asking them to provide their own in a box. |  | *"Sometimes it can be hard to identify what we need - especially when we're taking care of a baby and not thinking about ourselves. You can find inspiration below:    Today I need:   To take a shower and use the bathroom alone  To stay home all day  To have an hour to myself  To talk to a friend  To have physical contact (e.g. a hug)  To let my partner know that my body feels different and that I need time  To spend some time with my older child"* |  |
| Mothers should know that it takes effort to go through iCARE (as therapy does) and should not be discouraged despite experiencing difficulties | Add text to note that effort is needed just as in other therapies and to be understanding of oneself when encountering difficulties in introduction module |  | *" your own efforts are important - just like in regular treatment. You may find treatment challenging or confrontational, and it may bring up feelings such as sadness, frustration or anxiety. We recommend that you see this as part of the treatment and be forgiving of yourself."* |  |
|  | Remind importance of practice |  | *"Paying attention to what we're doing takes practice."* |  |
| Offer flexibility and advice on how find time to go through the intervention | Additional advice on how to make time for iCARE and ask for support |  | *"Take some time to think about how and when you can make time for the program. You can also consider whether it might be a good idea to tell a good friend, partner or family member about your treatment, for example."* |  |
| Retention is important as early as possible | Encouragement added to prevent early drop-out |  | *"On the next page you will be introduced to how the treatment works. Stay tuned as we explain how to use the program and get the most out of it. "* |  |
| Text-heavy content can be a burden | Minimal deletions or reduction of text but included more interaction and audios | *"In the next step, you will start working with your thoughts, which are related to how you feel. Now we will focus on your actions, which are easier to change."* | Deleted to avoid repetition |  |
|  |  | *"One strategy to disengage from negative thoughts is to increase distancing yourself from your thoughts and simply observe them. This is harder said than done. The essence is that you have to come to the realization that thoughts are just thoughts, they can be false, harsh, irrelevant and that's perfectly normal."* | *"When doing self-care activities, difficult or negative thoughts or feelings may come and disturb you."*  Added an audio exercise to practice defusion |  |
| Avoid implying that negative circles are easy to identify or can be eliminated | Text taken out | *"congratulations on completing your first negative circle"* | Deleted |  |
| More interaction and repetition with the acquired knowledge | More multiple choice options added |  | *(check all that apply)*  *How do you think these thoughts might make the mother feel? (check all that apply)*  *Sad  Upset  Hopeless   How do you think these thoughts affect her body? (check all that apply)  Fatigue  Tension   How do you think these thoughts might make this mom take action?  By starting to do many things at once and not having time to rest.  By staying at home and isolating herself.   Can the mother analyze the situation in a different way? Yes, she can No"* |  |
| More encouragement for trying exercises | Encouraging sentences added throughout iCARE | *"However, it's still important to find some time to take care of ourselves, especially when we have symptoms of postpartum depression.   Postpartum depression can reduce our motivation and energy. Our negative thoughts and feelings can cause us to stay away from situations and activities that can help us."* | *"However, it's still important to find some time to take care of yourself, especially with symptoms of postpartum depression. It's also important that you keep doing it. You can think of self-care activities like watering a plant. It's important to keep watering the plant to keep it growing. Postpartum depression can reduce our motivation and energy. Our negative thoughts and feelings can cause us to stay away from situations and activities that can help us."*  *"It's normal to find it hard to refocus. Try to do it for just 5 minutes a day. And praise yourself for trying. "*  *"Will my difficult thoughts disappear after I do the exercises? No, they may come back.    But by practicing using your senses and distancing from negative thoughts (exercises), you will help yourself to focus more on the activity of self-care - making sure you take care of yourself and others. "*  *"Maybe this step is the most challenging for you, if so, take it a little at a time."* |  |
| Replicate micro feedback from talk therapies | Added to accompany mothers through iCARE |  | *"It can be hard to get started at first, but hang in there, it will get easier once you've practiced for a while”* |  |
| Acknowledge how difficult it can be to confront one's symptoms and expectations about motherhood | Encouraging working through the difficult parts |  | *"It can be difficult to accept that you have symptoms of postpartum depression and perhaps let go of some ideas about what motherhood is. Remember that you can stop in the program and reflect on what you are experiencing."* |  |
| Normalize feeling worse before feeling better | Emphasis on fluctuations rather than relapse |  | *"Be aware that the results of the questionnaire can go up and down, mood and symptoms can change in either direction during treatment. It takes time to get better."* |  |
|  |  | *"Relapse can happen at any time when working to reduce symptoms of postpartum depression."* | *"You can have a good day and the next day find yourself not feeling well again. These fluctuations are completely normal and can happen at any time when working to reduce symptoms of postpartum depression."* |  |
| Encourage symptom check as a self-assessment tool | Added that symptom check can be an indication, but only an indication |  | *"Using your score, you can get an idea of how your symptoms manifest on the postpartum depression scale. However, your symptom score is only an indication. It's up to you to assess how you feel and whether you need further help."* |  |
| Voice-over of all text | Option to have text read aloud with Microsoft Edge added (introduction step) |  | *"You can have the text in the treatment program read out if you are using the Microsoft Edge browser. To do this, click the icon next to the address bar, as shown below. If you would like to use the Microsoft read aloud feature, the voice package must be enabled under settings on your computer."* |  |
| Learning for visual materials needs repetition (videos with mom and baby) | Add each video two times and signal what we want mothers to pay attention to |  |  |  |
| Audio files appreciated, more audio files to help understanding of exercises and practice on the go | Audio clips added in modules 1, 2 and 4.  Reminder to use audio exercises |  | *"You can listen to this audio clip if you want to practice unplugging from difficult thoughts while doing your self-care activities:"* |  |
| Answers to exercises not only in writing | Text changed to encourage doing the exercises in different ways (introduction step) | *"If you don't want to type anything in the text field, insert an "x"."* | *"We recommend you to write in the text fields as it is part of the treatment, but it should not be a burden for you. You can also choose to write down your answers on paper/in your own diary/on post-its, audio record your answers or say them aloud to others. If you don't want to write anything in the text field, insert an 'x'."* |  |
| **Inclusivity and gender representation** | | | | |
| Emphasize shared responsibility between mother and partner | Language revised | *"You might also consider telling a good friend, partner or family member about your treatment, for example…Maybe they can look after the child/children while you go through your steps."* | *"… Maybe they can spend time with your baby while you go through your steps and exercises or help you stick to the program."* |  |
|  |  |  | *"Families are different and distribute tasks and care differently between them. Either way, it's important that you feel supported in the task of being there for your child."* |  |
| Use parents instead of mothers and fathers |  | *"Some moms and dads quickly form a close emotional bond with their baby right after birth, but most parents need more time to form a close relationship with the baby and develop an emotional bond."* | *"Some parents quickly form a close emotional bond with their baby right after birth, but most parents need more time to form a close relationship with the baby and develop an emotional bond. "* |  |
| The colors in the beginning are gender stereotypical (referring to the pink and purple first shot of the videos) | No change as videos would need to be edited and this comment came from only one participant |  |  | No changes. Will be reviewed during the feasibility study |
| Illustrations should include more family constellations | No changes now. Comment came from one participant |  |  | More illustrations will be included after the feasibility study |
| **Clarity of instructions and data use** | | | | |
| It should be clear that the research group contacts a mother if symptoms worsen |  |  | *"The research team observes your results and in case of high fluctuation, you will be contacted.* |  |
| Clearer who can see the written answers and what they are used for |  | *"Only you and the healthcare professionals behind the program can see what you write. What you write will not be disclosed" to others.* | *"Only you and the researchers behind the study can see what you write in the text fields.* Your answers are used by the research group in anonymized form and to evaluate the interaction with and effect of the program. Your doctor and nurse cannot see what you write." |  |
| It should be clear that mothers can do the exercises when they are ready. | Encouraging sentences added to make clear that users can stop and return to exercises when they feel ready. | *"Between each step, we suggest you work on the exercises regularly to feel the effects of the program as much as possible.*" | *"Between each step, we suggest that you work on the exercises regularly to feel the effects of the program as much as possible. Allow yourself to stop and come back if you find that something is difficult." (Introduction step)* |  |
| Mothers should know until when they have access to iCARE | Added until when access ceases |  | *"You have access to the iCARE platform and your answers until the research project ends (planned for December 2025)."* |  |
| Illustration of modules and overview of modules cannot be seen at the same time | Illustration changed to include the titles of the modules (introduction module) |  |  |  |
| "PDF" (for partners) is not a descriptive name and is hard to find | Changed to "Information for partner and relatives"  Placed in catalog |  |  |  |
| Subtitles should be slower in the first video with Maria | Changed to more subtitles at a time so they appear for longer |  |  |  |
| **Understanding of therapeutic method** | | | | |
| Cognitive therapy as the basis of iCARE should be conveyed clearer | Added that intervention uses cognitive therapy | *"You may recognize yourself in some of the symptoms described."* | *"An effective treatment for postpartum depression is cognitive therapy. This program is based on cognitive therapy, which can be understood from the cognitive diamond. In this step you will be introduced to the cognitive diamond."* |  |
|  |  | *"When you focus on postpartum depression, you work to understand and change the elements of the diamond that we can control ourselves, such as thoughts and behaviors. That's why we dive a bit more into what thoughts are like during postpartum depression."* | *"In cognitive therapy, you work to understand and change the elements of the diamond that you can control. These are thoughts and behavior. That's why we delve a little more into what thoughts are like during postpartum depression."* |  |

| **Table S4. thematic analysis from prototype testing with women with lived experience of PPD** | | | | | | | |
| --- | --- | --- | --- | --- | --- | --- | --- |
| **Theme** | **Subtheme** | ***Quote*** | **Interview 1 or 2** | **Participant** | **Suggested Change** | **Status** | **Additional Notes** |
| **Appropriateness of Content** | Illustrations & color tone | *Skeptical about colors at first, but in intro video they worked well… not bleak.* | 1 | W4 |  |  |  |
|  | Illustrations & color tone | *light purple palette—prefer more professional for confidence.* | 1 | W3 | Remove pacifier; tone down color palette | Implemented | Pacifier removed; colors under feasibility review |
|  | Illustrations emotionally appropriate | *Tones/colors are fine… not gloomy, not happy-go-lucky.* | 1 | W2 |  |  |  |
|  | Illustrations emotionally appropriate | *Illustrations fit where you are in life… great illustrations.* | 1 | W5 |  |  |  |
|  | Intro video brevity | *Nice little video… but boring—I don’t have much patience.* | 1 | W5 | Keep intro videos very short; allow skip | Deferred until next phase |  |
|  | Intro video tone/length | *Super concrete and you feel welcome… short and edible.* | 1 | W5 |  |  | Affirms acceptability |
|  | Remove pacifier from transitions | *I have a bit of a problem with that graphic with that pacifier… it puts focus on the child… could be provocative.* | 1 | W2 | Remove pacifier and keep focus on the mother | Implemented |  |
|  | Remove pacifier from transitions | *Who came up with the pacifier?… it doesn’t all have to be about having a baby.* | 1 | W4 | Remove pacifier icon in transitions | Implemented |  |
|  | Risk-factor video tone | *Risk-factor intro sounds like ‘your own fault’… makes me angry.* | 1 | W3 | Cut/reorder content of video to avoid blame; start with neutral factors | Deferred until next phase | Video changes postponed to pilot phase |
|  | Scope of program and fit | *If mild/moderate, program fits; ensure referral if needs are severe.* | 2 | W6 | Reiterate scope; strengthen referral pathways | Implemented | Reinforce referral pathways and need of in-person support for women with severe symptoms |
|  | Second video tone & production | *Acoustics were quite bad… harder to focus.* | 1 | W4 | Improve audio quality; reduce distracting gestures | Deferred until next phase | Video changes postponed to pilot phase |
|  | Second video tone & speaker | *one speaker doesn’t look into the camera… you don’t feel contact; the other is very present* | 1 | W2 | Warmer tone; presenter looking into camera; simpler language | Deferred until next phase | Video changes postponed to pilot phase |
|  | Examples can trigger worries and intrusive thoughts | *Examples at end of Step 1 could trigger intrusive thoughts.* | 2 | W4 | Review examples | Implemented partially | Re-test in pilot phase |
|  | Use “difficult/self-critical” thoughts | *‘Handle your negative thoughts’ provokes me—prefer just ‘thoughts’.* | 1 | W5 | Soften to “difficult/self-critical thoughts” | Implemented partially | Re-test in pilot phase |
|  | Mother stories (audios) unhelpful | *I’d rather have focus on myself than be disturbed by other people’s stories.* | 1 | W4 | Remind audio stories are optional | Implemented | Supports optionality/toolbox framing |
|  | Compassionate, non-stigmatizing language | *…praised for the first step… not being alone… detabooing language.* | 1 | W2 |  |  | Affirms language approach |
| **Motivation and Engagement** | Advice on making time & pacing | *Recommendation frequency is ambiguous… if there is a best way, write it clearly (‘we recommend X…’).* | 1 | W2 | State clear weekly cadence recommendation with flexibility | Implemented | Wording tweak to existing pacing guidance |
|  | Allow skipping ahead (non-research version) | *Would like to jump ahead to step 3.* | 1 | W3 | Enable step-jumping in future non-research release | Deferred until next phase | Review in pilot phase |
|  | Audio-first design | *Audio clips are good—can listen while caring for baby/walking; videos are difficult due to time.* | 1 | W5 | Prioritize audio; keep videos small | Implemented partially | Audio clips exist; extend where feasible |
|  | Emphasize own pacing | *Okay to pause, return, or jot keywords if overwhelmed.* | 2 | W2 | Reassurance is ok to pause and return | Implemented | Reduce dropout risk |
|  | Guidance for complex exercises | *Cognitive restructuring more complex—suggest guidance for difficult tasks.* | 2 | W2 | Add tip: break down steps; suggest partner support | Implemented partially | Re-test in pilot phase. Simplify restructuring exercise |
|  | Guided reflection prompts | *Self-reflection field too open… add optional guiding questions.* | 1 | W3 | Provide clickable prompts for structured reflection | Implemented | Implemented in some exercices |
|  | Positive experience exercise | *Positive experience every day helped focus on small good things.* | 2 | W4 | Keep exercise; ensure examples remain concrete |  | Affirms utility and acceptability |
|  | Self-care check-ins | *Follow-up check-ins on self-care list would be helpful (what you did/how it felt).* | 1 | W5 | Add short follow-up prompts. Reminders about self-care in next modules | Implemented |  |
|  | Structure & pacing | *Logical, pedagogical structure; easy navigation; videos short.* | 2 | W6 | Maintain current structure |  | Affirms usability |
|  | Symptom check as progress tracking | *Questionnaires let you see if it helps… follow getting better.* | 1 | W4 | Maintain mood tracking |  | Affirms supportive framing |
|  | Symptom check as progress tracking | *Symptom checks feel like pressure to get better… but can help you see improvement over waves.* | 1 | W5 | Frame as supportive tracking; normalize fluctuations | Implemented |  |
|  | Symptom check as progress tracking | *Symptom checks rewarding; weekly cadence makes sense.* | 2 | W6 | Keep weekly guidance; emphasize fluctuations |  |  |
|  | Video motivational tone | *Videos motivate and convey care; easier than text when stressed.* | 2 | W2 | Keep videos short; maintain motivational tone |  | Affirms acceptability and usability videos |
|  | Video redundancy | *Those little videos, that is, they don't do anything special for me. Because there isn't really anything other than what you read anyway. But. And it can be.* | 2 | W3 | Ensure videos add unique value; allow skip; provide concise text | Deferred until next phase |  |
|  | When to do exercises | *Confronting difficult feelings alone could be hard; suggest doing exercises when partner is present.* | 2 | W3 | Add tip: choose moments with support available | Implemented |  |
| **Inclusivity and Gender Representation** | Audio exemplars | *Audio clips were really good; could be even more examples.* | 2 | W2 | Include more short audio stories | Partially implemented | No more stories but add an extra mindfulness exercise |
|  | Audio exemplars | *Audio clips helpful; would like more (but keep brief).* | 2 | W6 | Include more short audio stories | Partially implemented | No more stories but add an extra mindfulness exercise |
|  | Color palette inclusivity | *Colors too stereotypically gender-normative… we should do something else.* | 1 | W5 | Adopt less gendered palette | Partially implemented | Some new graphs added |
|  | Right representation in audios | *Very different in the 2 examples, and It's also really good, I think….So this thing about It can, so we can end up in the same place in many different ways. I think the diversity reflects that...Immediate thoughts that It's good they are different the 2.* | 1 | W2 |  |  |  |
| **Clarity of Instructions and data use** | Ability to edit answers | *You can’t go back and correct?… allow edits or addendum notes.* | 1 | W4 | Permit addendum with timestamp (no deletion) | Partially implemented | Data can be seen at the end of the module and modified. Old data is lost |
|  | Ability to edit answers | *Allow adding to previous entries with a new timestamp (no deletion).* | 1 | W3 | Enable diary addendum with timestamps | Not possible |  |
|  | Data access transparency & monitoring | *That there must be an attempt to create trust here, i.e. that this information will not be shared with anyone….It does not pass on to your own doctor and health care…I understand that, but I was immediately in doubt as to why the health professionals who sit behind the program, why they should be able to see what I write. So you could perhaps write that in if that's what they do with that data...It is, so it could be a potential false security or an extra help that is not there. And I think it's very important that you don't think that.* | 1 | W2 | Clarify who sees the data, how data is used, who monitors datra | Implemented | EPDS data monitored every other day. |
|  | Data privacy statement | *Fear info could be shared with municipality—explicit ‘no third-party sharing’.* | 1 | W3 | Add explicit no-third-party sharing notice | Implemented |  |
|  | Emergency contacts access | *Sidebar/profile section with emergency numbers would be nice.* | 1 | W4 | Visible emergency contacts in each module | Implemented | Catalog list where to call included at the end of each module |
|  | Monitoring clarity & action steps | *Monitoring reminders could be even more explicit; if overwhelmed, call GP/health nurse/emergency.* | 1 | W5 | Visible emergency contacts in each module | Implemented | Catalog list where to call included at the end of each module |
|  | Monitoring expectation | *If I filled it in, I’d hope someone had seen it and I’d get help…* | 1 | W4 | Remind who checks EPDS | Implemented | Clarify that only will be contacted if EPDS above 18 or question 10 affirmative |
|  | Monitoring expectation | *If I write suicidal thoughts, will anyone respond? Clarify no backend monitoring; only self-referral.* | 1 | W3 | Remind that the call is not therapy, is to assess risk and refer to services | Implemented |  |
|  | Optionality cues | *Add text like ‘okay to skip or return later.’* | 2 | W2 | Insert reassurance text on skip/return | Implemented |  |
|  | Optionality cues | *Only knew I could skip writing because interviewer said so.* | 2 | W4 | Explain you can write X and skip exercise | Implemented |  |
|  | Proofreading | *Minor linguistic issues (double negations).* | 2 | W6 | Resolve typos and grammar | Implemented |  |
|  | Symptom checker purpose | *Symptom checker can feel confrontational; clarify purpose (self-evaluation + safety).* | 2 | W2 | Add explicit text clarifying purpose | Implemented |  |
|  | Symptom checker timeframe | *Add prompt like ‘past week’.* | 2 | W3 | Specify reference period consistently | Implemented |  |
|  | Symptom questionnaires purpose | *State that questionnaires are for research AND to help you follow your wellbeing.* | 1 | W2 | Explain dual purpose (research + self-tracking); emphasize indication-only | Implemented |  |
| **Promote Understanding of Therapeutic Method** | Text alternative to rationale video | *Prefer rationale in writing rather than video.* | 1 | W3 | Add concise text summary for each rationale | Deferred until next phase |  |
| **Usability / Layout & Navigation** | Overview collapse/expand | *Everything open feels unmanageable… better if only the section you’re in is expanded.* | 1 | W4 | Make shorter sections | Deferred until next phase | Reduce cognitive load |
|  | Navigation clarity (micro voice-over) | *A voice that says ‘move the mouse…’ would make it easier.* | 1 | W2 | Add brief voice-over cues to guide key UI actions | Platform not enabled for this change |  |
|  | Layout & Readability: Intro video pacing/subtitles | *If you don’t read fast, you might not understand without subtitles… maybe slow the pace.* | 1 | W3 | Ensure subtitles; slow pacing for readability | Implemented |  |
|  | Layout & Readability: Rebalance illustration vs instruction | *Quite a lot of illustration, very little text (symptom checker landing).* | 1 | W4 | Add clearer instructional text to checkpoints. Balance size graphics with text | Implemented |  |
|  | Layout & Readability: Reduce text burden | *Intro page mixes very different themes… And they had brains here think I can be quite tired, so if you can do it even more, cut it to the legs or actually stand it up in 2 slides just had to say, it could be that it could help it a little as you can see. These are different kinds of information.* | 1 | W2 | Split intro across screens; reduce cognitive load | Deferred until next phase |  |
|  | Mobile & Cross-Device: Layout balance (desktop vs phone) | *On computer illustrations look very large vs little text.* | 2 | W4 | Adjust desktop layout | Implemented |  |
|  | Mobile & Cross-Device: Phone-first experience & examples | *With the developed for the phone, that is, it worked better, I think the So format significantly much better.* | 2 | W3 | Prioritize mobile optimization |  | Affirms design |
|  | Navigation: Reduce scrolling / co-locate content | *Hassle to scroll up/down to connect illustration with text.* | 1 | W5 | Co-locate illustration and text or add anchors | Deferred until next phase |  |
|  | Structure & navigation | *Logical flow; easy navigation.* | 2 | W2 | Maintain current structure |  | Affirms usability |
|  | Video performance | *Videos a bit slow to load across different networks.* | 2 | W4 | Optimize streaming; provide audio/text fallback | Deferred until next phase |  |
| **Notes.** Interview 1 was a think aloud exercise, Interview two was a semi-structured interview after mothers tried the program during three weeks. | | | | | | | |
